# Biological Evaluation, Molecular Docking, and in Ovo Hatchability Assessment of Selected Mannich-Type 1,2,4-Triazol-5-one Derivatives

**DOI:** 10.3390/ijms27156948

**Published:** 2026-08-02

**Authors:** Songül Ulufer Bulut, Özlem Durna, Songül Boy, Fevzi Aytemiz, Gül Özdemir Toraman, Önder Albayrak, Murat Beytur, Ahmet Harmankaya, Haydar Yüksek, Gültekin Yildiz

**Affiliations:** 1 Pharmacy Services Department, Kağızman Vocational School, Kafkas University, Kars 36700, Türkiye; 2 Animal Nutrition and Nutritional Diseases Department, Veterinary Faculty, Dicle University, Diyarbakır 21280, Türkiye; odurna36@gmail.com; 3 Medical Services and Techniques Department, Atatürk Health Services Vocational School, Kafkas University, Kars 36000, Türkiye; songulboy36@hotmail.com (S.B.); fevziaytemiz53@gmail.com (F.A.); onder_albayrak024@hotmail.com (Ö.A.); 4 Department of Chemistry, Faculty of Science and Letters, Kafkas University, Kars 36000, Türkiye; gulozd91@gmail.com (G.Ö.T.); muratbeytur@kafkas.edu.tr (M.B.); ahmetharmankaya@kafkas.edu.tr (A.H.); hhigh61@gmail.com (H.Y.); 5 Animal Nutrition and Nutritional Diseases Department, Veterinary Faculty, Ankara University, Kars 06100, Türkiye; gyildiz@ankara.edu.tr

**Keywords:** 1,2,4-triazol-5-one derivatives, Mannich bases, molecular docking, in ovo, hatchability, antioxidant-related activity, metal chelation, antimicrobial activity

## Abstract

1,2,4-Triazole derivatives are widely investigated as bioactive heterocyclic compounds with diverse biological properties; however, their potential effects on avian embryonic development remain insufficiently characterized. This study aimed to synthesize selected Mannich-type 1,2,4-triazol-5-one derivatives and evaluate their in vitro biological activities, predicted enzyme interactions, and in ovo hatchability outcomes in broiler embryos. Three derivatives (**3a**–**3c**) were synthesized and structurally characterized using Fourier-transform infrared (FT-IR), proton nuclear magnetic resonance (^1^H-NMR), and carbon-13 nuclear magnetic resonance (^13^C-NMR) spectroscopy. Their reducing capacity, 2,2-diphenyl-1-picrylhydrazyl (DPPH) radical-scavenging activity, metal-chelating capacity, and antimicrobial activity were evaluated. Molecular docking analyses were performed against carbonic anhydrase II and catalase, using acetazolamide and 3-amino-1,2,4-triazole as reference inhibitors, respectively. For the in ovo assessment, fertilized Ross 308 eggs were allocated to non-injected control, vehicle control, and compound-treated groups. Compounds **3a**, **3b**, and **3c** were administered on incubation day 17 at 20, 10, and 5 mg/egg, respectively, as literature-supported, compound-specific exploratory exposure levels. The compounds exhibited weak reducing capacity, negligible DPPH radical-scavenging activity, pronounced metal-chelating capacity, and selective antimicrobial activity against several bacterial strains, whereas no inhibition was observed against *Escherichia coli*. Docking analysis predicted favorable interactions with both enzyme targets, with compound **3c** showing the most favorable binding energies. Baseline egg weights were comparable among groups, indicating that initial egg weight was unlikely to influence hatchability outcomes. Complete hatch failure occurred in all compound-treated groups, whereas hatchability was observed in the control groups. These findings suggest that the tested derivatives may adversely affect embryonic development under the applied in ovo conditions. Their pronounced metal-chelating capacity, together with the predicted interactions with carbonic anhydrase II and catalase, provides a plausible mechanistic framework for interpreting the observed embryonic outcomes. The molecular docking results complement the experimental findings and provide supportive in silico evidence for these potential interactions. These findings contribute to the limited literature on the in ovo evaluation of synthetic Mannich-type 1,2,4-triazol-5-one derivatives and provide preliminary evidence regarding their embryotoxic potential. Further dose-dependent, mechanistic, histopathological, and enzyme-based studies are required to clarify the biological basis of the observed effects and to guide future evaluation of these compounds in in ovo, biomedical, and veterinary applications.

## 1. Introduction

1,2,4-Triazole and 1,2,4-triazol-5-one derivatives have attracted considerable attention due to their diverse biological activities and their suitability for structure-based computational evaluation. Previous studies have reported the synthesis, spectroscopic characterization, molecular docking analysis, and biological evaluation of structurally related triazole derivatives, supporting the relevance of this scaffold in bioactive compound development [1,2]. Nitrogen-containing heterocyclic compounds are known to exhibit in vitro antibacterial and antifungal [3], antiparasitic [4], and antioxidant activities [5]. In particular, triazole-based Schiff bases have been reported to enhance cell membrane permeability through complex formation with transition metals, resulting in pronounced inhibitory effects [6,7]. Consequently, understanding how structural modifications influence the biological behavior of triazole derivatives has become an important research focus [8,9]. Collectively, these findings highlight 1,2,4-triazole derivatives as promising scaffolds for the development of biologically active compounds with potential applications in both human and veterinary medicine.

Beyond cellular-level investigations, these biological effects have also been demonstrated in in vivo pharmacological studies. In rodent models, 1,2,4-triazole derivatives have been shown to increase the activities of superoxide dismutase, catalase, and glutathione peroxidase, while significantly reducing oxidative stress [10,11]. Although previous pharmacological studies have demonstrated the biological potential of 1,2,4-triazole derivatives, most available evidence originates from rodent models, and biological responses may vary according to structural features and species-specific characteristics [10,11,12]. The predominance of rodent-based data and the limited availability of systematic information for avian species highlight a significant knowledge gap. Therefore, alternative experimental approaches are required to evaluate the biological and toxicological responses of triazole derivatives in poultry embryos.

The in ovo feeding technique enables the direct administration of nutrients and bioactive compounds during embryogenesis, providing physiologically relevant data for pharmacological and toxicological research [13,14]. Antioxidant compounds such as vitamin E, vitamin C, sodium selenite, and nanocurcumin have been reported to improve immunity, hatchability, and post-hatch performance when administered via this method [15,16]. However, studies investigating the in ovo application of triazole derivatives remain very limited. In a recent study, Durna et al. [17] evaluated the in ovo administration of the synthetic compound 1-(morpholin-4-yl-methyl)-3-(p-chlorobenzyl)-4-(4-hydroxybenzylideneamino)-4,5-dihydro-1*H*-1,2,4-triazol-5-one, and complete hatch failure was observed in the experimental group. This outcome was associated with the strong metal-chelating capacity and weak antioxidant activity of the compound. These findings suggest that the embryonic effects of 1,2,4-triazole derivatives may be influenced by structural features, highlighting the need for comparative evaluation of structure-dependent biological and embryotoxic responses.

Previous studies have shown that certain carbohydrates and amino acids may induce embryonic mortality following in ovo administration, and hatch failure is considered an inherent risk of this technique [18,19]. Moreover, in ovo responses are influenced by multiple factors, including compound concentration, injection volume, solvent properties, timing, and the developmental stage of the embryo [14,18,19,20,21]. Procedural factors associated with in ovo applications have also been reported to negatively affect hatchability [22,23,24,25]. Therefore, the interpretation of embryotoxic outcomes requires careful consideration of both compound-related and procedure-related factors. Standardized experimental conditions are therefore essential for distinguishing compound-specific biological effects from procedure-related influences. In this context, comparative evaluation of structurally related 1,2,4-triazole derivatives within a standardized in ovo experimental design may help clarify whether the observed hatchability outcomes are associated with compound-specific structural features and biological properties rather than procedural variability.

**Figure 1:**
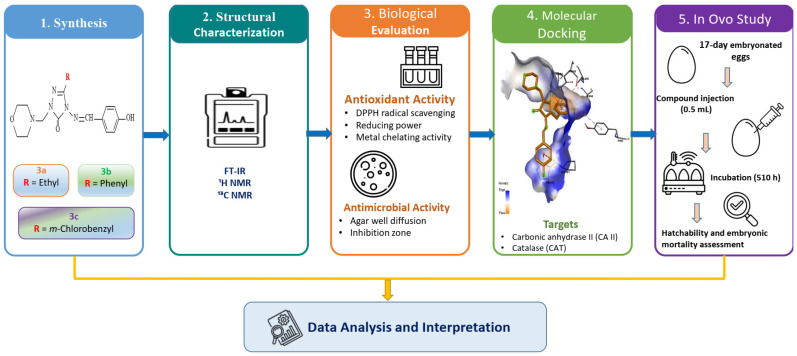
Schematic Overview of the Experimental Workflow.

Molecular docking analysis may further support the interpretation of compound-related biological effects by predicting possible interactions between synthesized derivatives and biologically relevant enzyme targets [26,27]. Recent structure-based studies have also applied docking, molecular dynamics, and virtual screening approaches to evaluate the target interactions of triazole-based compounds [28]. In the present study, carbonic anhydrase II and catalase were selected as docking targets due to their distinct biological roles in metal-dependent enzymatic processes and redox-related cellular defense. Carbonic anhydrase II is a zinc-containing metalloenzyme involved in CO_2_/bicarbonate conversion and pH homeostasis [29,30], whereas catalase is a heme-containing enzyme that catalyzes the decomposition of hydrogen peroxide into water and oxygen, thereby contributing to cellular redox homeostasis [31]. Since metal-chelating compounds may interact not only with toxic metal ions but also with essential metal ions and metal-dependent biological systems, such interactions may have dual biological implications [32,33]. Given the presence of multiple nitrogen and oxygen donor atoms in the synthesized Mannich-type 1,2,4-triazol-5-one derivatives, molecular docking analysis was performed to explore their possible interactions with these enzyme systems [34,35]. Despite the extensive investigation of triazole derivatives, studies evaluating their effects using in ovo models remain scarce [17]. The overall experimental design of the present study, including compound synthesis, structural characterization, biological evaluation, molecular docking, and the in ovo experimental approach, is summarized in Figure 1.

Accordingly, the primary aim of this study was to synthesize and characterize three selected Mannich-type 1,2,4-triazol-5-one derivatives and to evaluate their in vitro biological activities, molecular docking profiles, and in ovo hatchability outcomes in broiler embryos. The study was designed as a preliminary compound-specific screening to investigate whether structurally different Mannich derivatives exhibit adverse effects on hatchability at low, intermediate, and high exposure levels of 5, 10, and 20 mg/egg, respectively. In addition, antioxidant-related, metal-chelating, antimicrobial, and molecular docking analyses were performed to provide supportive biological and in silico data for interpreting the observed in ovo outcomes. Since the tested compounds differ in both structural features and selected screening doses, the study was not intended to establish a direct dose–response relationship, but rather to provide preliminary comparative insight into the hatchability-related embryotoxic potential of structurally related Mannich-type 1,2,4-triazol-5-one derivatives.

## 2. Results

### 2.1. Chemistry

**Scheme 1:**
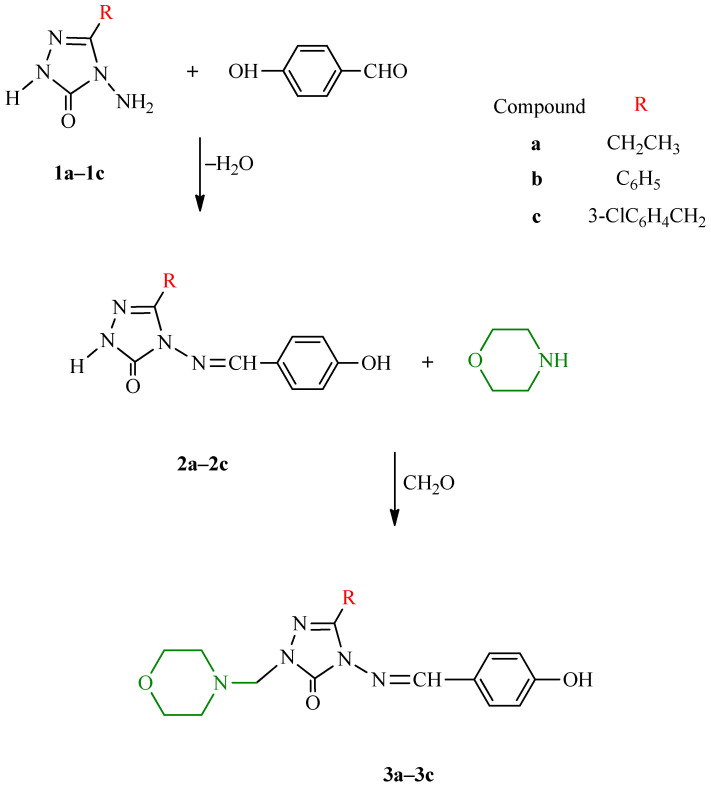
Synthetic route for Mannich-type 1,2,4-triazol-5-one derivatives **3a**–**3c**.

The synthetic route for the Mannich-type 1,2,4-triazol-5-one derivatives **3a**–**3c** is presented in Scheme 1. The 3-alkyl/aryl-4-amino-4,5-dihydro-1*H*-1,2,4-triazol-5-one precursors **1a**–**1c** were first converted into the corresponding Schiff base derivatives **2a**–**2c** by condensation with 4-hydroxybenzaldehyde. Subsequently, compounds **2a**–**2c** underwent Mannich reaction with morpholine and formaldehyde to afford the target Mannich base derivatives **3a**–**3c**. The target compounds were obtained in good yields and were coded according to the substituent at the 3-position of the 1,2,4-triazol-5-one ring: **3a** bearing an ethyl group, **3b** bearing a phenyl group, and **3c** bearing an m-chlorobenzyl group. The structures of the synthesized compounds were confirmed by Fourier-transform infrared (FT-IR), proton nuclear magnetic resonance (^1^H-NMR), and carbon-13 nuclear magnetic resonance (^13^C-NMR) spectroscopic analyses.

The FT-IR, ^1^H-NMR, and ^13^C-NMR analyses confirmed the proposed structures of compounds **3a**–**3c**, and the obtained spectroscopic data were consistent with those reported for structurally related Mannich-type 1,2,4-triazol-5-one derivatives. Detailed FT-IR, ^1^H-NMR, and ^13^C-NMR spectral data, together with the corresponding spectra, are provided in the Supplementary Materials (Tables S1–S3 and Figures S1–S9).

The FT-IR spectra showed the characteristic absorption bands of the phenolic OH, triazol-5-one carbonyl, and azomethine (C=N) groups, confirming the successful formation of the target Mannich-type derivatives. Likewise, the ^1^H-NMR and ^13^C-NMR spectra exhibited the expected signals corresponding to the morpholine ring, Mannich methylene group, aromatic substituents, azomethine carbon/proton, and triazole ring carbons. Overall, the spectroscopic findings consistently supported the proposed structures of compounds **3a**–**3c** prior to biological evaluation.

### 2.2. In Vitro Antioxidant Activity

Following structural confirmation, the in vitro antioxidant-related activities and metal-chelating capacities of the synthesized Mannich-type 1,2,4-triazol-5-one derivatives **3a**–**3c** were evaluated to obtain preliminary biological data prior to in ovo administration. These assays were performed to assess the redox-related behavior and metal-binding potential of the compounds, which may influence their biological effects under embryonic conditions. The reducing power of compounds **3a**–**3c** was evaluated at three different concentrations by spectrophotometric measurement at 700 nm.

**Figure 2:**
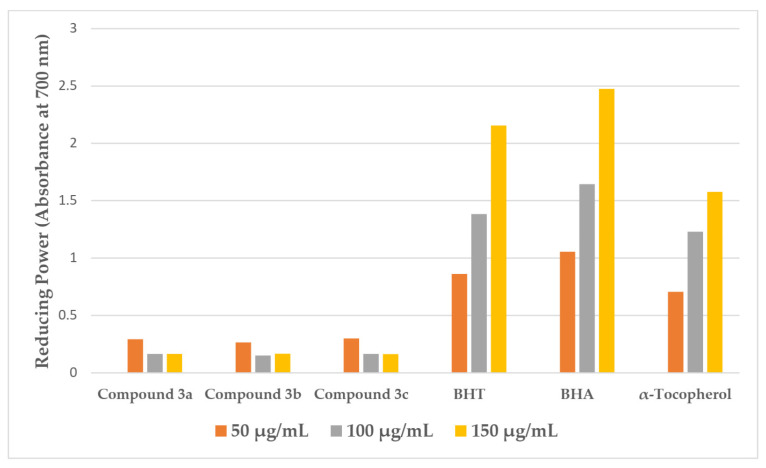
Reducing power activities of compounds **3a**–**3c**.

As illustrated in Figure 2, compounds **3a**–**3c** exhibited markedly lower reducing power than the standard antioxidants butylated hydroxytoluene (BHT), butylated hydroxyanisole (BHA), and α-tocopherol at all tested concentrations. The absorbance values of compounds **3a**–**3c** remained low and showed no concentration-dependent increase, whereas the reducing capacities of the standard antioxidants increased progressively with increasing concentration. These findings indicate that the synthesized Mannich-type 1,2,4-triazol-5-one derivatives exhibited only weak electron-donating/reducing capacity compared with the reference antioxidants, indicating minimal antioxidant activity through this mechanism.

**Figure 3:**
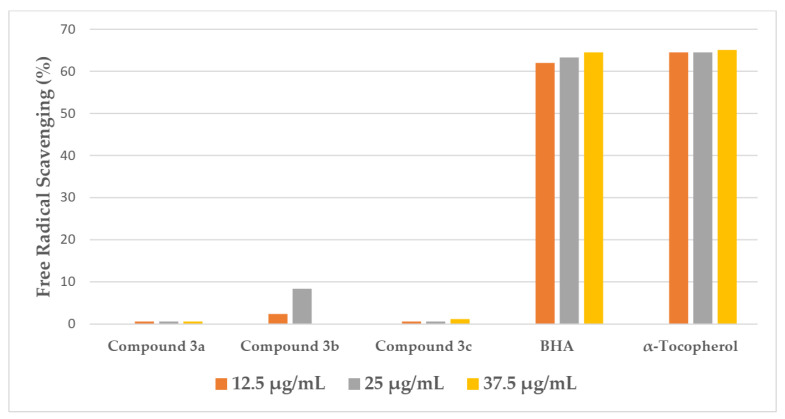
Free radical scavenging activities (%) of compounds **3a**–**3c**.

The 2,2-diphenyl-1-picrylhydrazyl (DPPH) radical-scavenging results are presented in Figure 3. The standard antioxidants BHA and α-tocopherol demonstrated consistently high radical-scavenging activity, with values above 60% at all tested concentrations. In contrast, compounds **3a**–**3c** showed very low and variable DPPH radical-scavenging activity. This finding indicates that compounds **3a**–**3c** exhibited no meaningful DPPH radical-scavenging activity under the tested conditions. The very low scavenging values compared with BHA and α-tocopherol suggest that these derivatives have insufficient hydrogen atom- or electron-donating capacity for effective direct radical neutralization.

**Figure 4:**
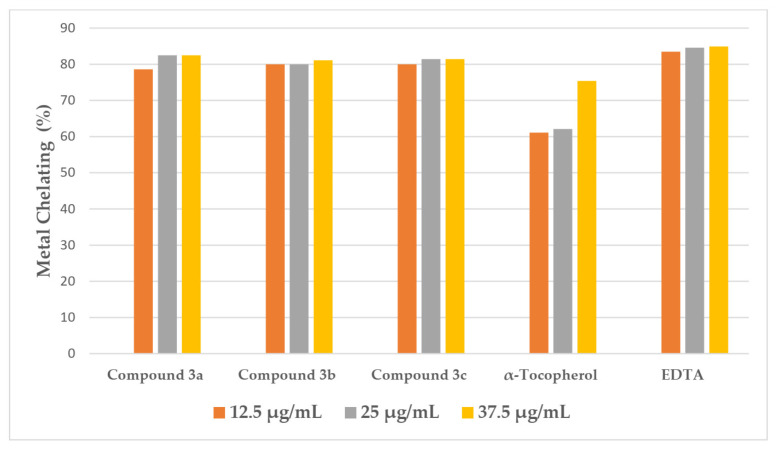
Metal chelating activities (%) of compounds **3a**–**3c**.

The metal-chelating assay results shown in Figure 4 demonstrated that compounds **3a**–**3c** maintained high chelating activity across the tested concentration range. A slight increase was observed between 12.5 and 25 µg/mL; however, the values changed only minimally between 25 and 37.5 µg/mL. Therefore, the compounds did not exhibit a pronounced concentration-dependent increase in metal-chelating activity but rather showed an early plateau, with consistently high activity at the tested concentrations. In comparison, α-tocopherol showed lower chelating activity, whereas ethylenediaminetetraacetic acid (EDTA) displayed the highest activity among the reference compounds. These findings indicate that compounds **3a**–**3c** possess marked metal-binding capacities, with chelating activities approaching those of EDTA under the tested conditions. This pronounced chelating ability may be associated with the presence of nitrogen and oxygen donor atoms within the triazole, Schiff base, and morpholine-related structural moieties, which may facilitate coordination with metal ions. In contrast to their weak reducing power and negligible DPPH radical-scavenging activity, the pronounced metal-chelating behavior of these compounds indicates that their metal-binding capacity is more prominent than their direct antioxidant activity. Such a profile may have dual biological implications: while metal chelation may limit metal-mediated oxidative processes, excessive or nonspecific metal binding may also interfere with essential metal ions and metal-dependent enzymes, potentially contributing to adverse biological effects under in ovo conditions.

### 2.3. In Vitro Antimicrobial Activity

The antimicrobial activities of compounds **3a**–**3c** against selected bacterial strains are summarized in Table 1. In this preliminary agar well diffusion screening, all three compounds produced measurable inhibition zones against selected Gram-positive and Gram-negative bacteria, whereas no inhibition zone was observed against *Escherichia coli*. Among the tested derivatives, compound **3b** produced the largest inhibition zones, measuring 24 mm against *Staphylococcus aureus* and 23 mm against *Bacillus subtilis*, *Bacillus cereus, Klebsiella pneumoniae*, and *Pseudomonas aeruginosa*. Compound **3a** also produced relatively large inhibition zones, particularly against *Bacillus subtilis* and *Pseudomonas aeruginosa*, whereas compound **3c** showed variable inhibition zone diameters depending on the bacterial strain.

Although ampicillin generally produced larger inhibition zones than the synthesized compounds, based on inhibition zone diameters obtained under the present assay conditions, compounds **3a**–**3c** produced zones comparable to or larger than those of *neomycin* and *streptomycin* against several tested microorganisms. The absence of activity against *E. coli* may be associated with the outer membrane barrier characteristic of Gram-negative bacteria, which can restrict the penetration of certain bioactive compounds [37]. Overall, these findings indicate that compounds **3a**–**3c** produced a selective pattern of bacterial growth inhibition under the present agar well diffusion conditions, with compound **3b** yielding the largest inhibition zones among the synthesized derivatives.

These antimicrobial findings provide additional supportive evidence that the synthesized derivatives can interact with biological systems; however, they should not be directly interpreted as a mechanism underlying the observed in ovo hatchability outcomes.

### 2.4. Molecular Docking Results

Molecular docking analysis showed that compounds **3a**–**3c** exhibited favorable predicted binding affinities toward both carbonic anhydrase II and catalase (Table 2).

In Table 2, among the tested derivatives, compound **3c** displayed the strongest predicted binding affinity for both carbonic anhydrase II (−9.7 kcal/mol) and catalase (−10.8 kcal/mol). Compound **3b** also showed notable binding affinities, particularly toward catalase (−9.9 kcal/mol), whereas compound **3a** exhibited comparatively lower but still favorable predicted interactions with both enzyme targets. To provide a reference for interpreting the docking results, acetazolamide and 3-amino-1,2,4-triazole were docked against carbonic anhydrase II and catalase, respectively, using the same computational protocol. In Table 2 the predicted binding energies of compounds **3a**–**3c** were therefore evaluated in relation to these reference inhibitors. Acetazolamide showed a predicted binding energy of −6.5 kcal/mol toward carbonic anhydrase II, whereas 3-amino-1,2,4-triazole showed a predicted binding energy of −4.4 kcal/mol toward catalase. Under the present computational conditions, compounds **3a**–**3c** exhibited more negative predicted binding energies than the corresponding reference inhibitors. However, these differences should not be interpreted as evidence of greater experimental inhibitory potency.

Following the binding energy analysis, compound **3c** was selected for detailed interaction analysis because it showed the strongest predicted binding affinity toward both target enzymes. The bond distances, detailed predicted binding modes, and ligand–protein interaction diagrams of compounds **3a** and **3b** with catalase and carbonic anhydrase II are provided in the Supplementary Materials (Tables S4 and S5). The corresponding detailed interaction profiles of the reference inhibitors acetazolamide and 3-amino-1,2,4-triazole with their respective target enzymes are presented in Table S6.

As shown in Table 3, in carbonic anhydrase II, compound **3c** formed conventional hydrogen bonds with ASN A62 and GLN A92, along with π-related and hydrophobic interactions involving HIS A94, HIS A96, PRO A201, PRO A202, TYR A7, and ALA A65. In catalase, compound **3c** showed a conventional hydrogen bond with ASN A148 and multiple π-related and hydrophobic interactions with residues such as PHE A153, PHE A161, TYR A358, GLY A353, ARG A354, VAL A73, and ALA A357. These interaction profiles may support the favorable predicted binding affinities of compound **3c** toward both enzyme targets.

In Table 3, the unfavorable bump observed between compound **3c** and THR A361 in the catalase complex represents a possible local steric clash. Although this interaction does not necessarily invalidate the overall predicted binding pose, it indicates that the local ligand–residue geometry may be suboptimal and reduces confidence in interpreting this pose as a fully favorable interaction.

**Figure 5:**
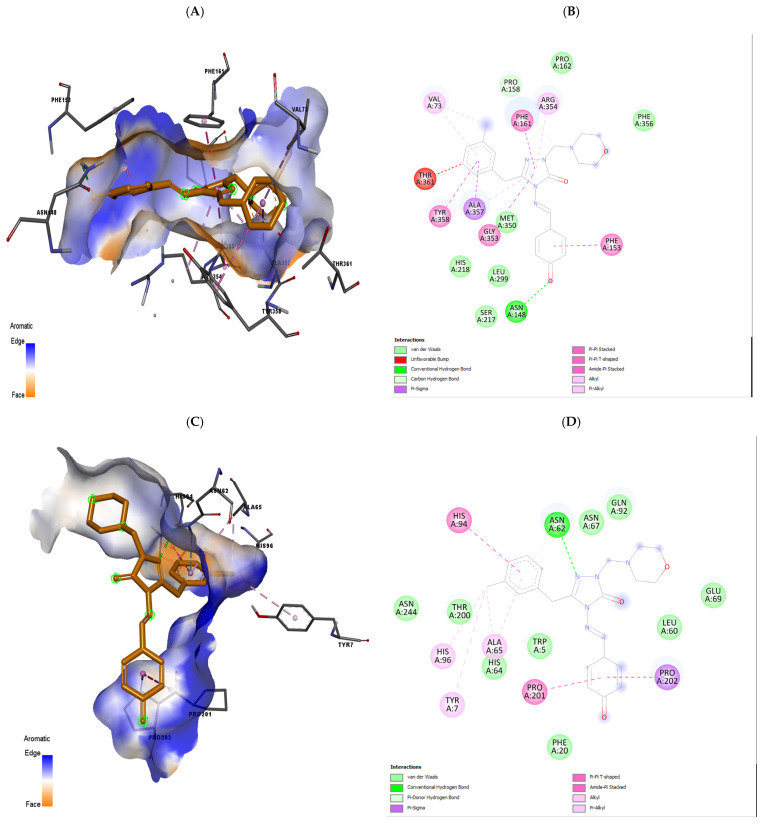
Predicted binding modes and interaction profiles of compound **3c** with catalase and carbonic anhydrase II. (**A**) Three-dimensional binding mode of compound **3c** in catalase (1DGF). (**B**) Two-dimensional interaction diagram of compound **3c** with catalase. (**C**) Three-dimensional binding mode of compound **3c** in carbonic anhydrase II (1BN1). (**D**) Two-dimensional interaction diagram of compound **3c** with carbonic anhydrase II.

The predicted binding modes and key interaction profiles of compound **3c** with carbonic anhydrase II and catalase are illustrated in Figure 5. 

Because compound **3c** showed the strongest predicted binding affinity toward both target enzymes, its predicted binding modes and key ligand–protein interactions with catalase and carbonic anhydrase II were further examined. The 3D binding poses and 2D interaction diagrams of compound **3c** are presented in Figure 5. These docking findings suggest that compound **3c** may have a higher potential to interact with enzyme systems associated with redox homeostasis, metal-dependent catalysis, and pH regulation. However, these results should be interpreted as supportive in silico evidence rather than direct proof of enzyme inhibition or antioxidant activity.

The corresponding predicted binding modes and interaction profiles of compounds **3a** and **3b** with both target enzymes are provided in the Supplementary Materials (Figures S10 and S11). The predicted binding modes and interaction profiles of the reference inhibitors 3-amino-1,2,4-triazole and acetazolamide with catalase and carbonic anhydrase II, respectively, are presented in Figure S12.

### 2.5. In Ovo Hatchability Outcomes

After the in vitro evaluation, the in ovo hatchability outcomes were analyzed. A total of 200 viable fertilized Ross 308 eggs were allocated into five experimental groups: control, dimethyl sulfoxide/phosphate-buffered saline (DMSO/PBS) vehicle, compound **3a**, compound **3b**, and compound **3c** groups, with 40 eggs in each group. Eggs were incubated under standard conditions, and in ovo administration was performed on day 17 of incubation under sterile conditions. Incubation was continued until 510 h, corresponding to the completion of the standard incubation period for broiler embryos.

Hatchability differed significantly among the five experimental groups according to the Fisher–Freeman–Halton exact test using Monte Carlo estimation with 10,000 sampled tables (*p* < 0.001). Hatchability was 82.5% in the control group and 75.0% in the DMSO/PBS vehicle group. In contrast, no hatchability was observed in any of the compound-treated groups, with 0.0% hatchability recorded for compounds **3a**, **3b**, and **3c**. Hatchability data for all groups are summarized in Table 4.

Break-out examination of all unhatched eggs confirmed the presence of dead embryos; therefore, these outcomes were classified as embryonic mortality.

### 2.6. Statistical Evaluation of Baseline Egg Weights

To clarify whether the observed hatchability differences were influenced by initial egg characteristics, baseline egg weights were further examined.

As summarized in Table 5, Levene’s test indicated that the assumption of homogeneity of variances was not met for baseline egg weights (*p* = 0.001). Therefore, Welch’s ANOVA was applied. The analysis showed no statistically significant difference in baseline egg weights among the five experimental groups (Welch’s F = 0.624, *p* = 0.647), indicating comparable initial egg weights prior to in ovo administration. This result was further supported by the Brown–Forsythe test (*p* = 0.641). Consistently, Games–Howell post hoc comparisons revealed no significant pairwise differences among the groups (*p* > 0.05).

## 3. Discussion

The successful synthesis and structural verification of compounds **3a**–**3c** provided a reliable chemical basis for interpreting the subsequent biological evaluations. Since the FT-IR and NMR data were consistent with the proposed Mannich-type 1,2,4-triazol-5-one structures, the observed in vitro antioxidant, metal-chelating, antimicrobial, and in ovo hatchability outcomes can be interpreted in relation to the intended structural features of the synthesized derivatives. The presence of ethyl, phenyl, and m-chlorobenzyl substituents at the 3-position of the triazol-5-one ring enabled a preliminary structure-related comparison within the same Mannich-type framework. However, because the study included a limited number of compounds and compound-specific exposure levels, these findings should be considered preliminary structure-related observations rather than a definitive structure–activity relationship.

In vitro analyses performed to evaluate the biological profiles of the structurally validated compounds **3a**–**3c** showed that these derivatives did not exhibit a unidirectional biological pattern. The weak reducing power and negligible free radical-scavenging activities observed for the tested compounds indicate minimal activity through classical antioxidant mechanisms, reflecting insufficient electron- or hydrogen-donating capacity. In contrast, the marked metal-chelating capacities observed for the compounds indicate a different mode of biological interaction. Although metal chelation may contribute to the suppression of metal-mediated free radical formation, excessive or strong metal-binding capacity may also interfere with metal-dependent biological processes. Therefore, this feature may represent both a potential biological advantage and a possible risk depending on the biological context.

Molecular docking analysis provided supportive in silico evidence for the possible interaction of compounds **3a**–**3c** with carbonic anhydrase II and catalase. Among the tested derivatives, compound **3c** exhibited the strongest predicted binding affinities toward both enzyme targets, suggesting a higher potential for interaction with metal-dependent and redox-related enzyme systems. This finding is consistent with the strong metal-chelating profile observed in the in vitro assays and supports the possibility that these compounds may interact with biologically important enzyme systems associated with metal ion balance and redox homeostasis. However, molecular docking results should not be interpreted as direct evidence of enzyme inhibition, antioxidant activity, or a confirmed mechanism of embryotoxicity.

The predicted catalase binding pose of compound **3c** included an unfavorable bump with THR A361, indicating a possible local steric clash. This interaction may reflect a locally suboptimal ligand orientation or a limitation of the rigid-receptor docking approach. Although the steric clash does not necessarily invalidate the overall predicted binding pose, it reduces confidence in interpreting the compound **3c**–catalase interaction as uniformly favorable. Therefore, the favorable calculated binding energy should be considered together with this local geometric limitation, and the predicted binding mode should be interpreted cautiously.

Recent studies provide methodologically heterogeneous experimental context for triazole–carbonic anhydrase II interactions. Medetalibeyoğlu et al. [38] reported human carbonic anhydrase II (hCA II) inhibition by an *N*-acetyl-derived 1,2,4-triazole Schiff base, with a half-maximal inhibitory concentration (IC_50_) value of 10.62 μM and an inhibition constant (K_i_) value of 11.72 μM. Aouad et al. [39] and Abd Al Moaty et al. [40] described stronger submicromolar inhibition by sulfonamide-tethered triazole hybrids and triazolo [4,3-a]pyrimidinone derivatives, respectively. These findings indicate that carbonic anhydrase II inhibitory activity varies considerably with scaffold design, substitution pattern, and incorporated pharmacophoric groups. However, IC_50_ values from different studies should not be directly ranked because assay conditions and experimental protocols may differ.

Quantitative docking studies provide additional, although non-equivalent, computational context. Virk et al. [41] reported a predicted binding affinity of −10.8791 kcal/mol for a 1,2,4-triazole derivative against bovine CA II, whereas Camadan et al. [42] reported −8.21 kcal/mol for a triazole–thiazole hybrid against hCA II. Çelik et al. [43] reported hCA II Glide scores of −3.785 to −4.667 kcal/mol for benzimidazole–triazole hybrids and −7.097 kcal/mol for acetazolamide. In the present study, acetazolamide and 3-amino-1,2,4-triazole were evaluated as reference inhibitors using the same docking protocol as compounds **3a**–**3c**, enabling an internal comparison under identical computational conditions. Comparisons with literature docking scores should nevertheless be interpreted cautiously because they depend on the protein structure, docking platform, preparation procedures, and scoring function employed.

The biological relevance of the selected reference inhibitors is supported by independent experimental evidence. High-resolution crystallographic analysis has demonstrated that acetazolamide binds directly to the active-site Zn^2+^ ion of human carbonic anhydrase II and forms additional interactions with key active-site residues [44]. In contrast, 3-amino-1,2,4-triazole is a well-established catalase inhibitor, and its mechanism-dependent inhibition involves covalent binding to the enzyme active center, a process that has been demonstrated experimentally in yeast [45]. Because this inhibitory mechanism differs from the non-covalent interactions evaluated by conventional molecular docking, its biological activity may not be fully represented by docking scores alone. Accordingly, although the predicted binding energies of compounds **3a**–**3c** and the reference inhibitors provide useful computational support for their potential interactions with carbonic anhydrase II and catalase, these findings should not be interpreted as direct evidence of enzyme inhibition or experimental potency. Rather, the docking results should be considered supportive in silico evidence that complements the experimental observations.

The pronounced metal-chelating capacity of the tested compounds may have dual biological implications. While metal chelation may limit metal-mediated oxidative processes, excessive or nonspecific metal binding may also interfere with essential metal ions and metal-dependent enzymes required for normal embryonic development. Therefore, the strong metal-chelating behavior, together with the predicted interactions with carbonic anhydrase II and catalase, may represent a possible contributing factor to the complete hatch failure observed under in ovo conditions. Nevertheless, since enzyme inhibition, oxidative stress markers, mitochondrial function, and metal-dependent enzyme activities were not directly measured, this interpretation should be considered a hypothesis requiring further experimental confirmation.

Antimicrobial evaluations demonstrated that compounds **3a**–**3c** exhibited selective inhibitory effects against the tested bacterial strains. The observed inhibition against Gram-positive bacteria such as *Staphylococcus aureus* and *Bacillus subtilis* suggests that these Mannich-type derivatives may interact with bacterial cell wall- or membrane-associated structures. Activity against Gram-negative bacteria, including *Klebsiella pneumoniae* and *Pseudomonas aeruginosa*, indicates species-dependent antimicrobial effects, whereas the lack of activity against *Escherichia coli* highlights variability in bacterial susceptibility. Although the reference antibiotics generally showed stronger or broader activity, the ability of the synthesized compounds to produce measurable inhibition zones in selected strains provides preliminary evidence of antimicrobial potential.

Nevertheless, inhibition zone diameters alone do not provide a definitive quantitative measure of antimicrobial potency. Agar diffusion results may be influenced by factors such as compound solubility, diffusion through the agar medium, molecular size, and physicochemical properties. Therefore, the present findings should be interpreted as preliminary evidence of antibacterial activity rather than as a direct comparison of antimicrobial potency. Future studies should determine minimum inhibitory concentration (MIC) and minimum bactericidal concentration (MBC) values using standardized broth dilution methods to confirm and quantitatively characterize the antimicrobial effects of compounds **3a**–**3c**.

These in vitro findings are also relevant for interpreting the in ovo outcomes, as compounds capable of interacting with microbial membranes, enzymes, or metal-dependent systems may also affect sensitive embryonic biological processes under specific exposure conditions.

The complete absence of hatchability in all compound-treated groups suggests that compounds **3a**–**3c** may adversely affect embryonic development under the applied in ovo conditions. When considered together with the in vitro and molecular docking data, this outcome may be partly associated with the biological profiles of the tested Mannich-type 1,2,4-triazol-5-one derivatives, which were characterized by negligible radical-scavenging activity, weak reducing capacity, pronounced metal-chelating ability, and predicted interactions with metal-dependent and redox-related enzyme systems. Such a profile may indicate a potential imbalance between antioxidant protection and metal-binding capacity, which could adversely affect metal-dependent biological processes during embryonic development. Excessive metal chelation may also interfere with the physiological availability of essential metal ions required for normal embryonic development. Zinc is indispensable for the activity of numerous zinc-dependent enzymes and transcription factors involved in DNA synthesis, cell proliferation, differentiation, and organogenesis. Consequently, excessive sequestration of zinc may impair these developmental processes. In addition, disturbances in metal homeostasis may indirectly affect calcium-dependent signaling pathways involved in embryonic tissue differentiation and morphogenesis [46,47,48,49]. During late embryogenesis, increased metabolic activity and oxygen demand may render embryos particularly vulnerable to disturbances in redox homeostasis and metal-ion balance. However, because oxidative stress markers, mitochondrial function, metal-dependent enzyme activities, and tissue-level alterations were not directly assessed, this interpretation should be considered a possible explanation rather than a confirmed mechanism. Although break-out analysis confirmed embryonic mortality in all unhatched eggs, the examination was performed solely to verify embryonic death and did not establish its developmental timing, pathological characteristics, or specific cause.

In addition to compound-related biological properties, formulation and administration conditions should also be considered. The tested compounds were administered as suspensions rather than true solutions due to their limited aqueous solubility, which may have influenced local exposure and distribution within the amniotic environment. Furthermore, in ovo outcomes can be affected by multiple procedural parameters, including injection volume, vehicle composition, timing of application, and embryonic developmental stage. Therefore, the observed hatchability failure should be interpreted within an integrated framework that considers both the intrinsic properties of the Mannich-type 1,2,4-triazol-5-one derivatives and the conditions of in ovo administration.

The present findings are also consistent with the limited available evidence on synthetic triazol-5-one derivatives in avian embryos. Durna et al. [17] reported complete hatch failure following in ovo administration of a structurally related p-chlorobenzyl-substituted Mannich-type 1,2,4-triazol-5-one derivative. In the present study, complete hatch failure was observed following the administration of ethyl-, phenyl-, and m-chlorobenzyl-substituted Mannich derivatives. This finding suggests that structurally related compounds within this scaffold may be associated with adverse hatchability outcomes under the tested in ovo exposure conditions. Nevertheless, because the tested compounds differed structurally and were administered at different compound-specific screening doses, these results should not be interpreted as evidence of a direct dose–response relationship or a definitive structure–activity relationship. Instead, they provide preliminary structure-related evidence supporting the need for further dose-dependent, mechanistic, and structure-focused investigations.

The absence of statistically significant differences in baseline egg weights among the five groups indicates that initial egg mass was not a confounding factor in the observed hatchability outcomes. Moreover, the maintenance of relatively high hatchability rates in the control and DMSO/PBS vehicle groups suggests that the general experimental procedure and vehicle system did not independently account for the complete hatch failure observed in the compound-treated groups. Therefore, the lack of hatchability in the **3a**-, **3b**-, and **3c**-treated groups may be related, at least in part, to exposure to the tested Mannich-type 1,2,4-triazol-5-one derivatives under the applied experimental conditions.

Future studies should include direct enzyme inhibition assays for carbonic anhydrase II and catalase to verify the biological relevance of the docking predictions. In addition, the assessment of oxidative stress biomarkers, metal homeostasis, mitochondrial function, and histopathological alterations in embryonic tissues would provide further insight into the mechanisms underlying the observed embryotoxicity. Such investigations would help clarify whether the pronounced metal-chelating activity of these compounds directly contributes to the impaired embryonic development observed in the present study.

## 4. Materials and Methods

### 4.1. Ethical Approval and Compliance

This study was approved by the Kafkas University Animal Experiments Local Ethics Committee under separate approval codes for each experimental protocol (KAÜ-HADYEK/2019-082, KAÜ-HADYEK/2019-084, KAÜ-HADYEK/2019-088, approval date: 22 May 2019). All experimental procedures were conducted in accordance with national regulations governing the care and use of laboratory animals and complied with Directive 2010/63/EU of the European Parliament on the protection of animals used for scientific purposes.

The experimental design was structured in line with the 3R principles (Replacement, Reduction, Refinement), and the number of experimental units was limited to ensure scientific validity while minimizing animal use.

### 4.2. Chemicals, Synthesis and Structural Characterization

All chemicals used were of analytical grade and were purchased from Sigma-Aldrich (St. Louis, MO, USA), Merck (Darmstadt, Germany), and Fluka (Buchs, Switzerland). FT-IR spectra were recorded using a Bruker ALPHA-P FT-IR spectrometer (Bruker Optik GmbH & Co. KG, Ettlingen, Germany). ^1^H-NMR and ^13^C-NMR spectra were obtained on a Bruker AVANCE III 400 spectrometer (Bruker BioSpin, Fällanden, Switzerland) operating at 400 MHz for ^1^H-NMR and 100 MHz for ^13^C-NMR at the Mersin University Central Research Laboratory. Melting points were determined using a WRS-2A microprocessor (INESA, Shanghai, China) melting point apparatus.

The 3-alkyl/aryl-4-amino-4,5-dihydro-1*H*-1,2,4-triazol-5-one precursors **1a**–**1c** were synthesized according to a previously reported Pinner-based method [50]. The corresponding Schiff base derivatives **2a**–**2c** were obtained by the condensation of compounds **1a**–**1c** with 4-hydroxybenzaldehyde. In the general procedure, the appropriate 3-alkyl/aryl-4-amino-4,5-dihydro-1*H*-1,2,4-triazol-5-one derivative **1a**–**1c** (0.01 mol) was dissolved in acetic acid (20 mL), and 4-hydroxybenzaldehyde (0.01 mol) was added. The reaction mixture was refluxed for 1.5 h and then cooled to room temperature. The precipitate formed upon the addition of distilled water was filtered, washed with distilled water, and dried under vacuum over CaCl_2_ in a desiccator. The crude product was recrystallized several times from ethanol and dried under vacuum to afford the Schiff base derivatives **2a**–**2c**.

The target Mannich base derivatives **3a**–**3c** were synthesized from the corresponding Schiff base derivatives **2a**–**2c** through a Mannich reaction with morpholine and formaldehyde. In the general procedure, the appropriate Schiff base derivative **2a**–**2c** (0.01 mol) was dissolved in ethanol (100 mL), followed by the addition of morpholine (0.015 mol) and 37% formaldehyde solution (0.02 mol). The reaction mixture was refluxed for 3 h, cooled to room temperature, and kept at −18 °C overnight. The resulting crude product was filtered, washed with cold ethanol, and dried. The obtained products were recrystallized several times from ethanol, dried under vacuum, and identified as the Mannich base derivatives **3a**–**3c**. Compound **3a** was obtained in 71% yield with a melting point of 96 °C, compound **3b** in 82% yield with a melting point of 189 °C, and compound **3c** in 73% yield with a melting point of 186 °C.

The target compounds were coded according to the structural nature of the substituent at the 3-position of the 1,2,4-triazol-5-one ring. Accordingly, compound **3a** was identified as 1-(morpholin-4-yl-methyl)-3-ethyl-4-(4-hydroxybenzylideneamino)-4,5-dihydro-1*H*-1,2,4-triazol-5-one, compound **3b** as 1-(morpholin-4-yl-methyl)-3-phenyl-4-(4-hydroxybenzylideneamino)-4,5-dihydro-1*H*-1,2,4-triazol-5-one, and compound **3c** as 1-(morpholin-4-yl-methyl)-3-(m-chlorobenzyl)-4-(4-hydroxybenzylideneamino)-4,5-dihydro-1*H*-1,2,4-triazol-5-one.

Compounds **3a**–**3c** were selected within the framework of the Pinner-based synthetic strategy to provide a preliminary evaluation of structurally distinct substituents while preserving the common Mannich-type 1,2,4-triazol-5-one scaffold [50]. Accordingly, ethyl (**3a**), phenyl (**3b**), and *m*-chlorobenzyl (**3c**) groups were selected to introduce differences in molecular size, aromaticity, lipophilicity, conformational flexibility, and electronic character. The ethyl group was included as a small electron-donating substituent with minimal steric demand, whereas the phenyl group was selected to introduce an aromatic system capable of potential π–π interactions. The *m*-chlorobenzyl substituent was chosen to combine the conformational flexibility of the benzyl linker with the electron-withdrawing and lipophilicity-enhancing effects of the chlorine atom. This limited series was intentionally designed as an exploratory compound set to examine how these structural variations might influence the biological properties of Mannich-type 1,2,4-triazol-5-one derivatives, rather than to establish a comprehensive structure–activity relationship.

Structural characterization of the synthesized compounds was carried out using FT-IR, ^1^H-NMR, and ^13^C-NMR spectroscopic techniques.

### 4.3. Antioxidant and Antimicrobial Activity Assays

The in vitro biological activities of the synthesized Mannich-type 1,2,4-triazol-5-one derivatives **3a**–**3c** were evaluated prior to the in ovo application. Antioxidant activity was assessed using reducing power, DPPH radical scavenging, and metal-chelating assays. Reducing power was measured at 700 nm according to the method of Oyaizu [51]. DPPH radical scavenging activity was determined at 517 nm following the method described by Blois [52]. Metal-chelating capacity was evaluated at 562 nm using Fe^2+^ ions according to the method of Dinis et al. [53]. α-Tocopherol, BHT, BHA, and EDTA were used as reference compounds.

Reducing power was expressed as absorbance at 700 nm, with higher absorbance values indicating greater reducing capacity. DPPH radical-scavenging and metal-chelating activities were calculated as percentages using Equation (1), where $A_{control}$ represents the absorbance of the control reaction and $A_{sample}$ represents the absorbance measured in the presence of the tested compound.

(1) $$\left\{\frac{A_{control}-A_{sample}}{A_{control}}\right\}\times 100$$

Antimicrobial activity was investigated using the agar well diffusion method [36] against three Gram-positive bacterial strains (*Staphylococcus aureus*, *Bacillus subtilis*, and *Bacillus cereus*) and three Gram-negative bacterial strains (*Klebsiella pneumoniae*, *Escherichia coli*, and *Pseudomonas aeruginosa*). Microorganisms were inoculated at 10^6^ CFU/mL on Mueller–Hinton agar plates. Solutions of compounds **3a**–**3c** at concentrations of 250–500 μg/mL were applied to the wells. DMSO was used as the negative control, whereas ampicillin, neomycin, and streptomycin were used as positive controls.

The antimicrobial evaluation was performed as a preliminary screening using the agar well diffusion method. The resulting inhibition zone diameters were used to identify the presence and relative extent of antibacterial activity under the tested conditions. MIC and MBC values were not determined, as quantitative characterization of antimicrobial potency was beyond the scope of the present study.

### 4.4. In Ovo Administration Protocol

In ovo administration is a technique based on the direct delivery of bioactive substances to the developing embryo during incubation and is used to evaluate embryonic physiological responses [14]. The success of this application depends primarily on the timing of injection, the administered volume, and the anatomical site of administration. In the present study, the amniotic sac was selected as the injection site, as administration into the amniotic fluid allows embryonic oral uptake and may directly influence early gastrointestinal exposure [22].

The optimal period for in ovo administration has been reported to be days 17–18 of incubation, when the amniotic fluid volume is sufficient for safe and effective delivery [54]. Applications performed during this developmental window have been shown to influence digestive system maturation and enzyme activity [23]. However, previous studies have also demonstrated that injection volumes exceeding 0.6 mL may negatively affect embryonic metabolism and delay or inhibit hatching [22]. Therefore, strict control of injection volume and adherence to sterile conditions are considered essential prerequisites for successful in ovo applications [14].

Owing to the limited aqueous solubility of the tested Mannich-type 1,2,4-triazol-5-one derivatives, the in ovo formulations were prepared as suspensions rather than true solutions. For each egg, the target amount of the relevant compound was placed separately into a sterile tube: 20 mg for compound **3a**, 10 mg for compound **3b**, and 5 mg for compound **3c**. Each compound was initially wetted with 3 µL of DMSO, resulting in a final DMSO concentration of 0.6% to improve particle wetting and dispersion. The final volume was then adjusted to 0.5 mL using sterile PBS (pH 7.3), selected for its physiological compatibility with embryonic tissues and its ability to maintain a stable ionic and pH environment [55]. Although PBS maintained the formulation pH, complete dissolution of the compounds was not achieved. Therefore, each egg-specific formulation was vigorously vortexed for 30–60 s immediately before injection to minimize sedimentation and promote a uniform distribution of suspended particles. Immediately after vortexing, the entire suspension was drawn into the syringe and administered to the assigned egg without prolonged standing. Sedimentation was not quantitatively assessed; however, because particle settling could occur if the formulation remained undisturbed, the short interval between vortexing, syringe loading, and administration was used to minimize sedimentation-related variability and promote consistent delivery of the intended dose within each treatment group.

In ovo injections were performed on day 17 of incubation using a sterile 21G needle (BD, Franklin Lakes, NJ, USA). A volume of 0.5 mL of the prepared suspension was delivered into the amniotic fluid via the air cell. Prior to injection, the eggshell surface was disinfected with ethanol, and after injection, the puncture site was sealed with sterile tape to reduce the risk of contamination. Broiler chicken embryos have a standard incubation period of approximately 21 days, corresponding to 504–510 h [56]; therefore, hatching was monitored until 510 h, representing the completion of the incubation period.

Injection volume is an important factor affecting embryonic development and hatchability in in ovo applications. Improper adjustment of injection parameters may disrupt embryonic metabolic balance and adversely affect hatching success. In addition, both the injected volume and the characteristics of the carrier medium have been emphasized as critical factors influencing embryo tolerance [14,24]. Within this framework, the 0.5 mL injection volume used in the present study was selected to minimize excessive physiological burden on the embryo and to remain within a volume considered suitable for in ovo applications performed during the late embryonic stage (E17–E19).

The doses used in in ovo applications are known to vary depending on the chemical nature, biological activity, solubility, day of injection, and intended physiological response of the administered substance. Previous studies have reported that doses within the range of 5–20 mg/egg have been used for different bioactive compounds in in ovo studies. Zhang et al. [57] administered chito-oligosaccharide and chlorella polysaccharide at 5 or 20 mg/egg on embryonic day 12.5 and observed changes in the cecal microbiota on day 21 post-hatch. Similarly, Faseleh Jahromi et al. [58] administered an oligosaccharide extract from palm kernel cake at 20 mg/egg and evaluated its effects on post-hatch general health and gut microbiota-related parameters. In ovo administration of prebiotics and synbiotics has also been investigated in terms of lymphocyte subsets in immune organs and immune responses [59]. In methisoprinol studies, 5, 10, and 20 mg/egg doses were administered in ovo to turkey eggs on day 26 of incubation; additionally, 5 and 20 mg/egg doses were evaluated for their effects on spleen morphology [60,61]. For vitamin E, Salary et al. [62] administered 15 and 30 mg/egg on embryonic day 14 and reported that in ovo vitamin E injection increased hatchability. Regarding synthetic triazole derivatives, Durna et al. [17] evaluated a p-chlorobenzyl-substituted Mannich-type 1,2,4-triazol-5-one derivative at 15 mg/egg for in ovo embryotoxicity and reported no hatchability in the experimental group. Taken together, these studies indicate that doses within the 5–20 mg/egg range have been used in in ovo preliminary screening studies. However, these doses should not be interpreted as a definitive safe dose range. Accordingly, in the present study, 5, 10, and 20 mg/egg were considered low, intermediate, and high exposure levels, respectively, and were used as compound-specific preliminary screening doses to evaluate the hatchability outcomes and embryotoxic potential of Mannich-type 1,2,4-triazol-5-one derivatives with different structural features. Since the tested compounds differ structurally, this classification was not intended to establish a direct dose–response relationship. The present study therefore adopted literature-supported exploratory exposure levels to enable the preliminary biological evaluation of structurally distinct Mannich-type 1,2,4-triazol-5-one derivatives under standardized experimental conditions.

The effectiveness of in ovo administration is influenced by multiple interacting parameters rather than by the chemical nature of the administered compound alone. In this context, factors such as dose, injection volume, site of administration, vehicle composition, and overall experimental conditions play an important role in determining embryonic responses. Therefore, the procedural framework applied in the present study was designed to minimize procedure-related variability and to support the interpretation of the observed hatchability outcomes in relation to the tested Mannich-type 1,2,4-triazol-5-one derivatives.

### 4.5. Incubation Conditions and in Ovo Application

The experiment was conducted at the Kafkas University Prof. Dr. Ali Rıza Aksoy Education, Research and Application Farm. Fertilized Ross 308 eggs were obtained from 35–42-week-old breeder hens. During incubation, the temperature and relative humidity were maintained at 37.7 °C and 60%, respectively, for the first 18 days, followed by 37.0 °C and 70% relative humidity for the final three days. On day 17 of incubation, viable embryos were identified by candling, and the eggs selected for in ovo application were marked.

For in ovo administration, three Mannich-type 1,2,4-triazol-5-one derivatives, compounds **3a**–**3c**, were evaluated within the same experimental design. Fertilized eggs were physically inspected, and those that were cracked, contaminated, or misshapen were excluded. After candling, 200 viable fertilized eggs were selected and allocated into five groups: a non-injected control group, a DMSO/PBS-treated vehicle group, a compound **3a**-treated group, a compound **3b**-treated group, and a compound **3c**-treated group, with 40 eggs assigned to each group. Compound **3a** was administered at 20 mg/egg, compound **3b** at 10 mg/egg, and compound **3c** at 5 mg/egg.

Accordingly, compounds **3a**–**3c** were administered at literature-supported, compound-specific exploratory exposure levels of 20, 10, and 5 mg/egg, respectively [17,57,58,59,60,61,62]. Because the derivatives were structurally distinct, the study was designed as a preliminary compound-specific screening rather than a standardized dose–response investigation. Therefore, the findings were not intended to establish a direct dose–response relationship or compare the relative potency of compounds **3a**–**3c**.

To ensure baseline homogeneity, eggs were allocated to the experimental groups according to their initial weights, resulting in comparable weight distributions among groups prior to in ovo administration. Weight-based allocation was applied during group assignment to minimize potential bias related to baseline egg weight. Following weight stratification, eggs were randomly assigned to the experimental groups. Each fertilized egg was considered as the experimental unit throughout the study. The sample size was set as 40 eggs per group based on similar in ovo studies reported in the literature and was considered sufficient for hatchability-based comparison among groups. No formal sample size calculation was performed. The group size was determined based on previous in ovo studies and practical incubation capacity. In ovo injection was performed on day 17 of incubation by delivering 0.5 mL of the prepared suspension into the amniotic fluid through the air cell using a sterile 21G needle. Prior to injection, the eggshell surface was disinfected with 75% ethanol, and the puncture site was sealed with sterile tape after the procedure.

Embryo injection was performed on day 17 of incubation only after the presence of a viable embryo had been confirmed by candling. Thereafter, the eggs were monitored by daily candling throughout the remainder of the incubation period. Hatchability was monitored until 510 h of incubation. At 510 h, all remaining unhatched eggs were re-examined by candling to identify embryos showing signs of internal or external pipping or readiness to hatch and eggs in which no evidence of hatching was observed. All unhatched eggs were subsequently subjected to break-out analysis by carefully opening the eggshell and examining the contents to confirm embryonic mortality. The break-out analysis was performed solely to verify the occurrence of embryonic death; no additional pathological or laboratory examinations were conducted to determine the specific cause or developmental timing of mortality.

Due to the nature of the experimental design, blinding was not applied. No humane endpoints requiring euthanasia were applied, as the study was limited to hatchability assessment as an indicator of embryonic response.

### 4.6. Molecular Docking Analysis

Protein and ligand structures were prepared using AutoDockTools (ADT) version 1.5.7. Molecular docking simulations were subsequently performed using AutoDock Vina 1.2.0 [63] to evaluate the predicted binding interactions of compounds **3a**–**3c** with the selected enzyme targets. Carbonic anhydrase II and catalase were selected as target proteins due to their biological relevance to metal-dependent enzymatic processes and redox-related cellular defense, respectively. Carbonic anhydrase II is a zinc-dependent metalloenzyme involved in CO_2_/bicarbonate balance and pH homeostasis, whereas catalase is a heme-containing enzyme responsible for the decomposition of hydrogen peroxide and the maintenance of redox balance [29,30,31]. The three-dimensional crystal structures of carbonic anhydrase II (PDB ID: 1BN1) and catalase (PDB ID: 1DGF) were retrieved from the Protein Data Bank and prepared as receptor models for docking studies.

The ligand structures of compounds **3a**–**3c** were prepared prior to docking analysis. Acetazolamide [44] and 3-amino-1,2,4-triazole [45] were included as reference inhibitors for carbonic anhydrase II and catalase, respectively. The reference inhibitors were prepared and docked using the same receptor preparation procedures, binding-site definitions, grid parameters, and computational protocol applied to compounds **3a**–**3c**. Docking simulations were carried out using the prepared ligand and receptor structures, and the resulting output files were generated for further analysis. The obtained docking poses, ligand–protein interaction profiles, interacting amino acid residues, interaction types, and bond distances were analyzed using BIOVIA Discovery Studio Client 2021. Docking results were expressed as binding energies in kcal/mol, and more negative values were interpreted as stronger predicted binding affinity. The docking findings were evaluated as supportive in silico data and were not considered direct evidence of enzyme inhibition or antioxidant activity.

### 4.7. Statistical Analysis

The primary outcome measure of the study was hatchability rate. Secondary outcomes included baseline egg weight comparisons and embryonic mortality. Hatchability and embryonic mortality rates were expressed as counts and percentages. Differences in hatchability among the five experimental groups were evaluated using the Fisher–Freeman–Halton exact test because the outcome variable was categorical. Monte Carlo estimation was used for exact significance testing when appropriate.

Baseline egg weights were statistically evaluated to assess homogeneity among the five experimental groups prior to in ovo administration. Variance homogeneity was assessed using Levene’s test. Since the assumption of homogeneity of variances was not met, Welch’s ANOVA was used to compare baseline egg weights among groups. Brown–Forsythe test was also considered as a robust test of equality of means. When appropriate, Games–Howell post hoc test was planned for pairwise comparisons. A *p*-value of <0.05 was considered statistically significant.

## 5. Conclusions

In conclusion, this study provides a preliminary evaluation of the in vitro biological activities, molecular docking profiles, and in ovo hatchability outcomes of three selected Mannich-type 1,2,4-triazol-5-one derivatives. The tested compounds exhibited negligible radical-scavenging activity, weak reducing capacity, marked metal-chelating ability, and selective antimicrobial activity against certain bacterial strains. Molecular docking analysis further indicated that compounds **3a**–**3c** may interact with carbonic anhydrase II and catalase, with compound **3c** showing the strongest predicted binding affinities toward both enzyme targets. The inclusion of acetazolamide and 3-amino-1,2,4-triazole as reference inhibitors provided additional context for interpreting the predicted binding energies; nevertheless, the docking findings should be regarded as supportive in silico evidence rather than direct proof of enzyme inhibition or antioxidant activity.

Under the applied in ovo conditions, complete absence of hatchability and confirmed embryonic mortality were observed in all compound-treated groups, whereas relatively high hatchability rates were maintained in the control and DMSO/PBS vehicle groups. This finding represents an important preliminary observation for the literature on synthetic triazol-5-one derivatives in avian embryos and suggests that exposure to compounds **3a**–**3c** may be associated with adverse effects on hatchability and embryonic development under compound-specific screening conditions.

However, these results should not be interpreted as evidence of a direct dose–response relationship, because the compounds differed structurally and were administered at different exposure levels. Similarly, the findings should be considered preliminary structure-related observations rather than a definitive structure–activity relationship. The possible contribution of negligible radical-scavenging and weak reducing capacities, pronounced metal-chelating ability, predicted interactions with metal-dependent and redox-related enzyme systems, suspension-based administration, and in ovo exposure conditions should be further investigated using mechanistic approaches. Future studies should include lower and fractionated doses, alternative injection volumes, different application timings, and direct measurements of oxidative stress markers, mitochondrial function, metal-dependent enzyme activities, enzyme inhibition profiles, and tissue-level alterations. Such studies will be important for defining safer exposure conditions and clarifying the developmental toxicology profile of Mannich-type 1,2,4-triazol-5-one derivatives in in ovo systems.

## Figures and Tables

**Table 1:** In Vitro Antimicrobial Activity Profiles of Compounds **3a**–**3c**.

	Microorganisms and Zone Diameter Values * (mm)					
	Gram-Positive			Gram-Negative		
Compounds	*Staphylococcus* *aureus*	*Bacillus* *subtilis*	*Bacillus* *cereus*	*Klebsiella* *pneumoniae*	*Escherichia* *coli*	*Pseudomonas* *aeruginosa*
**3a**	**19 (+++)**	**21 (+++)**	**18 (+++)**	**19 (+++)**	(−)	**20 (+++)**
**3b**	**24 (+++)**	**23 (+++)**	**23 (+++)**	**23 (+++)**	(−)	**23 (+++)**
**3c**	14 (++)	**20 (+++)**	14 (++)	**19 (+++)**	(−)	**21 (+++)**
*Ampisilin*	**37** **(+++)**	**33** **(+++)**	**36** **(+++)**	**35** **(+++)**	**34 (+++)**	**36 (+++)**
*Neomisin*	13 (++)	**17 (+++)**	**17 (+++)**	16 (++)	16 (++)	**17 (+++)**
*Streptomisin*	**21 (+++)**	12 (++)	12 (++)	11 (++)	10 (+)	12 (++)

* Antimicrobial activity was defined according to inhibition zone diameters: <5.5 mm (no activity, −), 5.5–10 mm (low, +), 11–16 mm (moderate, ++), and ≥17 mm (high, +++) [36]; bold values indicate high activity (≥17 mm).

**Table 2:** Molecular Docking Binding Energies of Compounds **3a**–**3c** and Reference Inhibitors Against Carbonic Anhydrase II and Catalase.

Carbonic Anhydrase II (1BN1)	Binding Energy (kcal/mol)
Acetazolamide	−6.5
Compound **3a**	−7.3
Compound **3b**	−8.2
Compound **3c**	−9.7
**Catalase (1DGF)**	**Binding Energy (kcal/mol)**
3-Amino-1,2,4-triazole	−4.4
Compound **3a**	−8.2
Compound **3b**	−9.9
Compound **3c**	−10.8

Acetazolamide and 3-amino-1,2,4-triazole were included as reference inhibitors for carbonic anhydrase II and catalase, respectively. All docking calculations were performed using the same computational protocol applied to compounds **3a**–**3c**.

**Table 3:** Key Predicted Ligand–Protein Interactions and Bond Distances of Compound **3c** with Carbonic Anhydrase II (1BN1) and Catalase (1DGF).

Target Enzyme	Interaction Type	Interacting Residue	Distance (Å)
Catalase (1DGF)	Conventional hydrogen bond	ASN A148	2.37
	Carbon hydrogen bond	PRO A158	3.32
	π–Sigma	ALA A357	3.75
	π–π stacked	PHE A153	3.97
	π–π stacked	PHE A161	3.79
	π–π T-shaped	TYR A358	5.10
	Amide π-stacked	GLY A353	4.91
	Alkyl	VAL A73	4.05
	π–Alkyl	ARG A354	4.51
	π–Alkyl	VAL A73	5.32
	π–Alkyl	ALA A357	4.42
	Unfavorable bump *	THR A361	1.85
Carbonic anhydrase II (1BN1)	Conventional hydrogen bond	ASN A62	2.42
	Conventional hydrogen bond	GLN A92	2.18
	π-Donor hydrogen bond	ASN A62	3.11
	π–Sigma	PRO A202	3.46
	π–π T-shaped	HIS A94	4.82
	Amide π-stacked	PRO A201	4.20
	Alkyl	ALA A65	3.76
	π–Alkyl	HIS A96	4.66
	π–Alkyl	TYR A7	2.47
	π–Alkyl	ALA A65	4.99

* It represents a possible steric clash rather than a favorable interaction.

**Table 4:** Hatchability and Embryonic Mortality Outcomes Among Experimental Groups.

Group	Number of Eggs	Injection Material	Dose/Final Concentration	Injection Volume (mL)	Hatched *n* (%)	Not Hatched Embryonic Mortality *n* (%)
Control	40	—	—	—	33 (82.5)	7 (17.5)
DMSO/PBS	40	DMSO	0.6% DMSO	0.5	30 (75.0)	10 (25.0)
Compound **3a**	40	Compound **3a** suspension	20 mg/egg	0.5	0 (0.0)	40 (100.0)
Compound **3b**	40	Compound **3b** suspension	10 mg/egg	0.5	0 (0.0)	40 (100.0)
Compound **3c**	40	Compound **3c** suspension	5 mg/egg	0.5	0 (0.0)	40 (100.0)

Note: Data are presented as counts and percentages. Group differences in hatchability were evaluated using the Fisher–Freeman–Halton exact test; *p* < 0.001.

**Table 5:** Baseline Egg Weight Comparisons Among Groups.

Group	Number of Eggs	Injection Material	Dose/Final Concentration	Injection Volume (mL)	Egg Weight Mean ± SD	Levene *p*-Value	Welch *p*-Value	Brown-Forsythe *p*-Value
Control	40	—	—	—	59.99 ± 4.02	*p* < 0.001	0.647	0.641
DMSO/PBS	40	DMSO	0.6% DMSO	0.5	60.11 ± 3.28			
Compound **3a**	40	Compound **3a** suspension	20 mg/egg	0.5	59.67 ± 2.49			
Compound **3b**	40	Compound **3b** suspension	10 mg/egg	0.5	60.84 ± 4.19			
Compound **3c**	40	Compound **3c** suspension	5 mg/egg	0.5	60.25 ± 2.77			

Note: Data are presented as mean ± SD. SD indicates standard deviation. Levene, Welch, and Brown–Forsythe *p*-values refer to the overall comparison among the five experimental groups.

## Data Availability

The original contributions presented in this study are included in the article. Further inquiries can be directed to the corresponding author.

## Supplementary Materials

The following supporting information can be downloaded at: https://www.mdpi.com/article/10.3390/ijms27156948/s1.
