# Supplementary material for: Biological Evaluation, Molecular Docking, and in Ovo Hatchability Assessment of Selected Mannich-Type 1,2,4-Triazol-5-one Derivatives
Source: Int J Mol Sci. 2026 Aug 2;27(15):6948. doi: 10.3390/ijms27156948 (PMC13467072; doi:10.3390/ijms27156948)
Supplement: Supplementary file 1 [file ijms-27-06948-s001.zip › ijms-4457202-supplementary.pdf]

Article

Biological Evaluation, Molecular Docking, and in Ovo Hatchability Assessment of Selected Mannich-Type 1,2,4-Triazol-5-one Derivatives

Songül Ulufer Bulut [1,*], Özlem Durna [2], Songül Boy [3], Fevzi Aytemiz [3], Gül Özdemir Toraman [4], Önder Albayrak [3], Murat Beytur [4], Ahmet Harmankaya [4], Haydar Yüksek [4] and Gültekin Yildiz [5]

1 Pharmacy Services Department, Kağızman Vocational School, Kafkas University, Kars 36700, Türkiye
2 Animal Nutrition and Nutritional Diseases Department, Veterinary Faculty, Dicle University, Diyarbakır 21280, Türkiye; odurna36@gmail.com
3 Medical Services and Techniques Department, Atatürk Health Services Vocational School, Kafkas University, Kars 36000, Türkiye; songulboy36@hotmail.com (S.B.); fevziaytemiz53@gmail.com (F.A.); onder_albayrak024@hotmail.com (Ö.A.)
4 Department of Chemistry, Faculty of Science and Letters, Kafkas University, Kars 36000, Türkiye; gulozd91@gmail.com (G.Ö.T.); muratbeytur@kafkas.edu.tr (M.B.); ahmetharmankaya@kafkas.edu.tr (A.H.); hhigh61@gmail.com (H.Y.)
5 Animal Nutrition and Nutritional Diseases Department, Veterinary Faculty, Ankara University, Kars 06100, Türkiye; gyildiz@ankara.edu.tr
* Correspondence: drsngulufer@kafkas.edu.tr

Table S1. FT-IR Spectral Data of Compounds **3a–3c.**

Compounds	Functional Group (ν, cm^{-1})			Aromatic Rings (ν, cm^{-1})		
	OH	C=O	C=N	1,4-disubstituted benzene ring	1,3-disubstituted benzene ring	Monosubstituted benzene ring
**3a**	3164	1694	1595	838	-	-
**3b**	3308	1678	1606, 1578	838	-	770 and 688
**3c**	3270	1700	1616, 1597	828	789 and 680	**-**

The FT-IR spectral data of compounds **3a–3c** supported the formation of the target Mannich-type 1,2,4-triazol-5-one derivatives. The broad absorption bands observed at 3164–3308 cm^{-1} were attributed to the phenolic ◎ H groups, while the carbonyl stretching vibrations of the triazol-5-one ring appeared at 1678–1700 cm^{-1}. The characteristic C=N stretching bands were detected at 1578–1616 cm^{-1}, indicating the presence of the azomethine structure. In addition, the aromatic ring vibration bands observed for the 1,4-disubstituted benzene ring and the substituent-specific aromatic systems were consistent with the proposed structures of compounds **3a–3c**.

Table S2. ^{1}H-NMR Spectral Data of Compounds **3a**–**3c** (δ, ppm; J, Hz).

Compounds	CH_3	CH_2CH_3	CH_2NCH_2	$ArCH_2$	CH_2OCH_2	NCH_2N	Aromatic H	N=CH	OH
**3a**	1.24 (t; J=7.20 Hz)	2.70 (q; J=7.20 Hz)	2.59 (t; J=4.40 Hz)	-	3.57 (t; J=4.40 Hz)	4.54 (s)	6.90 (d, 2H; J=8.80 Hz), 7.69 (d, 2H; J=8.80 Hz)	9.52 (s)	10.20 (s)
**3b**	-	-	2.67 (t; J=4.40 Hz)	-	3.60 (t; J=4.40 Hz)	4.70 (s)	6.91 (d, 2H; J=8.80 Hz), 7.54–7.57 (m, 3H), 7.69 (d, 2H; J=8.40 Hz), 7.91–7.94 (m, 2H)	9.43 (s)	10.28 (s)
**3c**	-	-	2.59 (t; J=4.40 Hz)	4.12 (s)	3.58 (t; J=4.40 Hz)	4.57 (s)	6.88 (d, 2H; J=9.20 Hz), 7.29–7.40 (m, 3H), 7.45–7.46 (m, 1H), 7.66 (d, 2H; J=8.80 Hz)	9.50 (s)	10.20 (s)

The ^{1}H-NMR spectral data of compounds **3a–3c** were consistent with the expected proton environments of the synthesized Mannich bases. The morpholine ring protons were observed as characteristic triplet signals corresponding to CH_2NCH_2 and CH_2◎ CH_2 groups, while the Mannich-type NCH_2N protons appeared as singlet signals at 4.54–4.70 ppm. The azomethine proton signals were detected at 9.43–9.52 ppm, and the phenolic ◎ H protons appeared at 10.20–10.28 ppm. The aromatic proton signals and substituent-specific aliphatic signals further supported the successful formation of the ethyl-, phenyl-, and m-chlorobenzyl-substituted derivatives.

Table S3. ^{13}C-NMR Spectral Data of Compounds **3a**–**3c** (δ, ppm).

Compounds	CH_3	CH_2CH_3	$ArCH_2$	CH_2NCH_2	NCH_2N	CH_2OCH_2	Aromatic C	Aromatic C (Linked to C-3)	Triazole C3	Triazole C5	N=CH
**3a**	9.79	18.46	-	49.89	66.12	66.15	115.83 (2CH), 124.24 (C), 129.72 (2CH), 160.78 (C)	-	146.76	150.50	155.29
**3b**	-	-	-	49.99	66.06	66.41	115.86 (2CH), 123.98 (C), 130.07 (2CH), 161.08 (C)	126.76 (C), 127.97 (2CH), 128.89 (2CH), 130.21 (CH)	143.12	150.62	158.33
**3c**	-	-	30.53	50.00	66.03	66.03	115.88 (2CH), 124.15 (C), 129.81 (2CH), 160.82 (C)	126.79 (CH), 127.48 (CH), 128.90 (CH), 130.30 (C), 132.96 (CH), 138.13 (C)	144.36	150.36	154.89

The ^{13}C-NMR spectral data further confirmed the proposed structures of compounds **3a–3c**. The carbon signals assigned to the morpholine ring, Mannich-type NCH_2N group, aromatic carbons, triazole C3 and C5 carbons, and azomethine carbon were observed in the expected regions. The substituent-related carbon signals, including the ethyl carbons in compound **3a** and the $ArCH_2$ carbon in compound **3c**, also supported the structural differentiation among the synthesized derivatives. ◎ verall, the ^{13}C-NMR data were in agreement with the FT-IR and ^{1}H-NMR findings and confirmed the successful synthesis of the target Mannich-type 1,2,4-triazol-5-one derivatives.

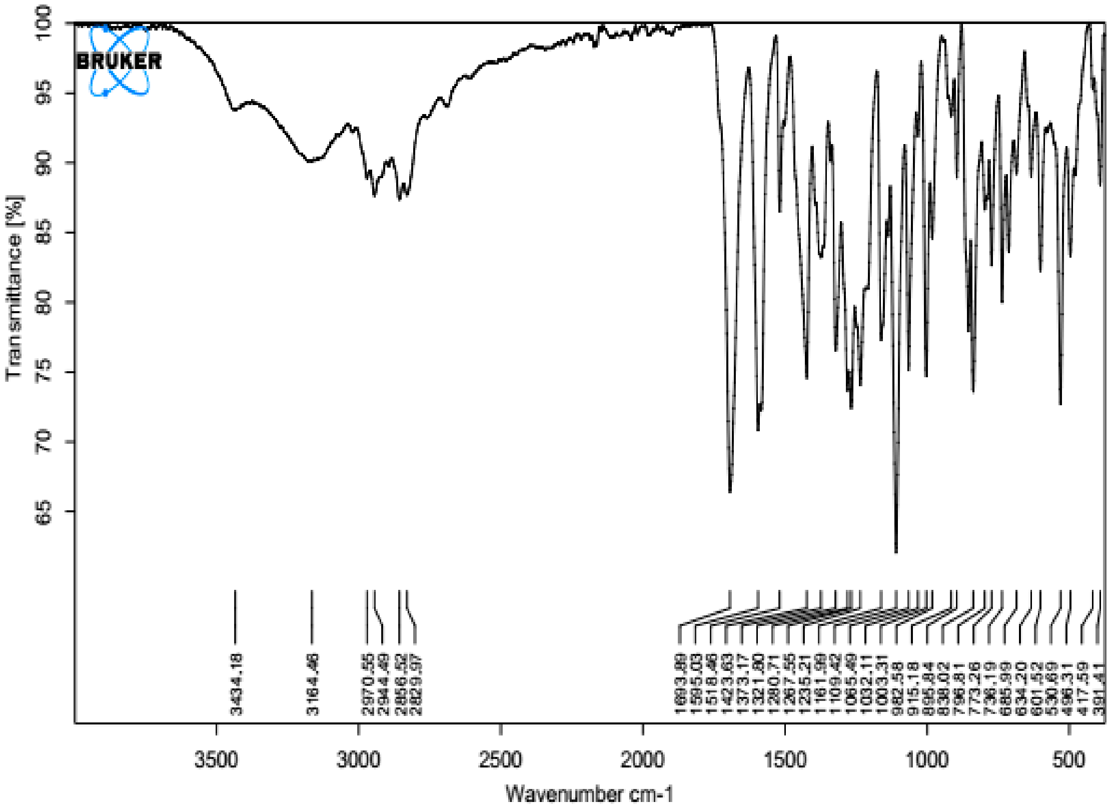

Figure S1. IR Spectrum of Compound 3a

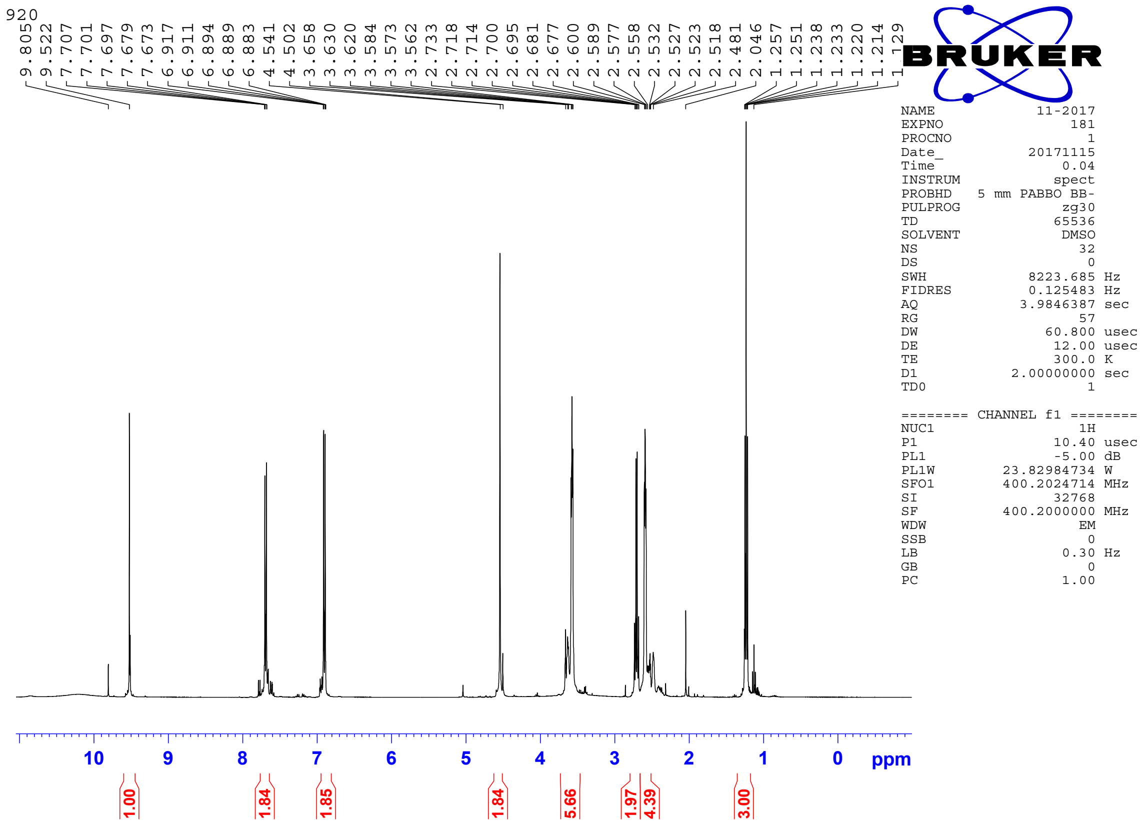

Figure S2. ^{1}H-NMR (DMS◎ -d₆) Spectrum of Compound 3a

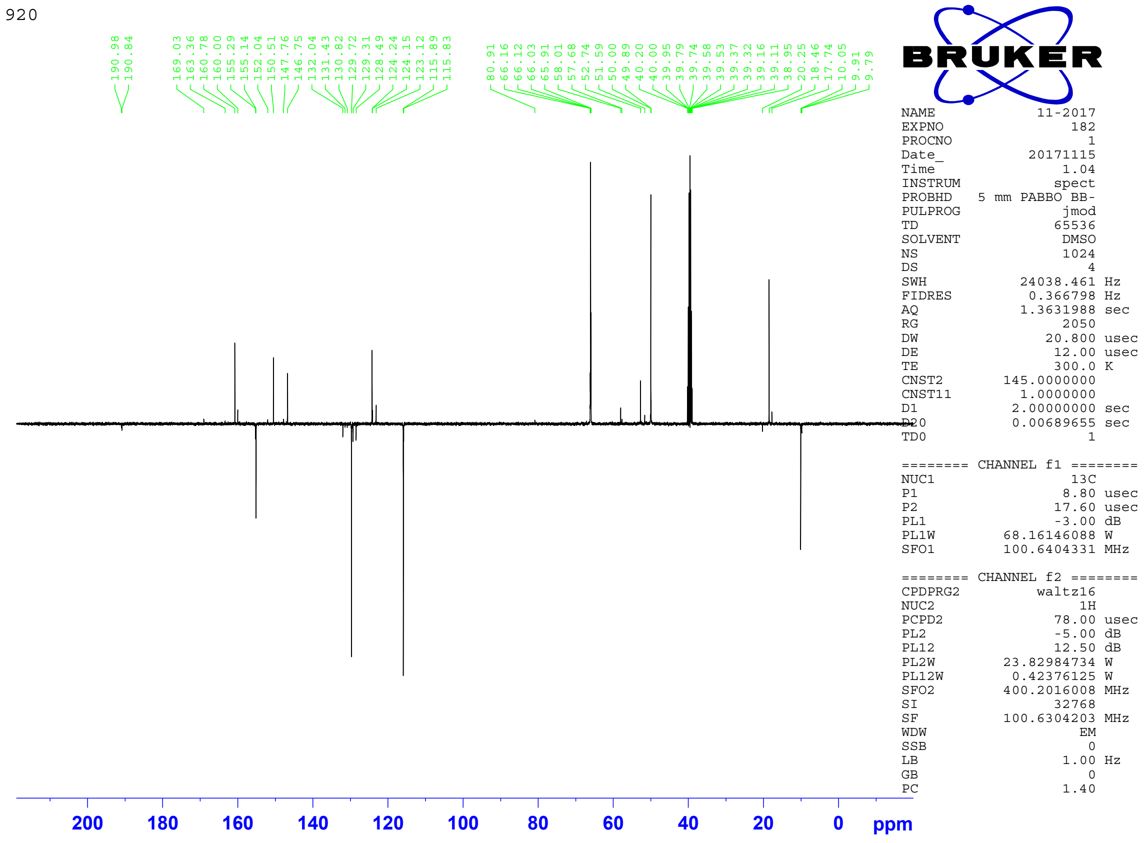

Figure S3. ^{13}C-NMR (DMSO-d_6) Spectrum of Compound 3a

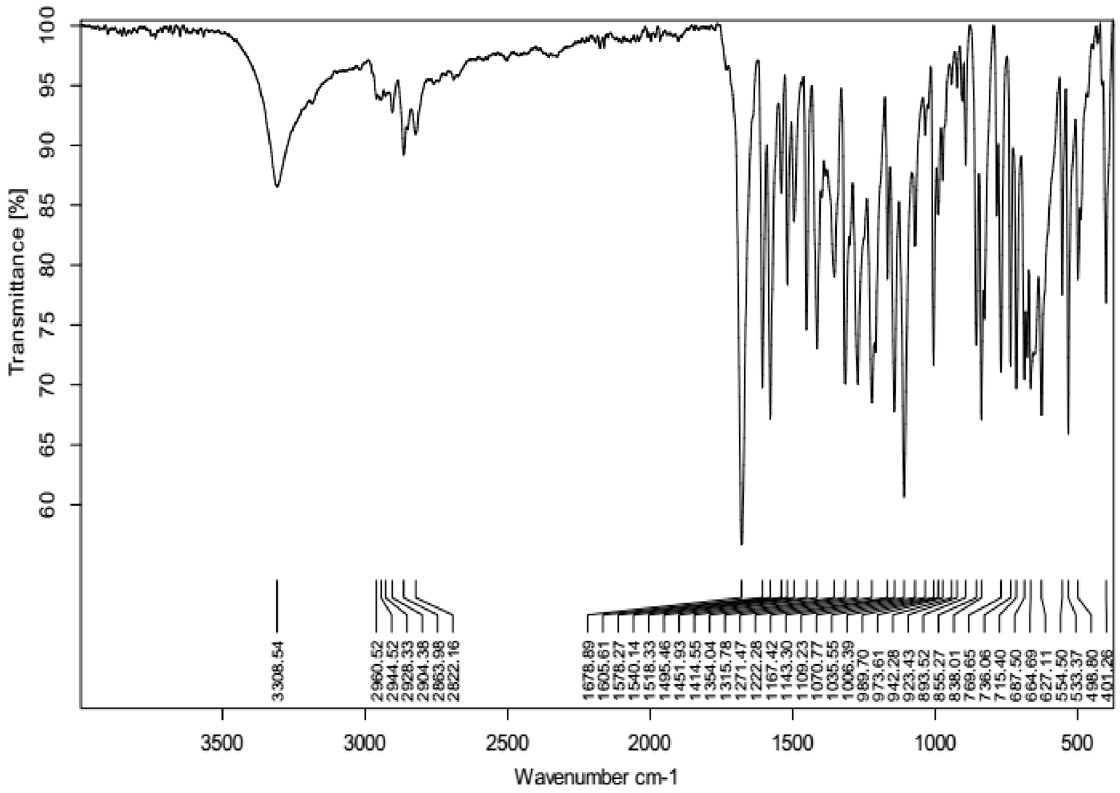

Figure S4. IR Spectrum of Compound 3b

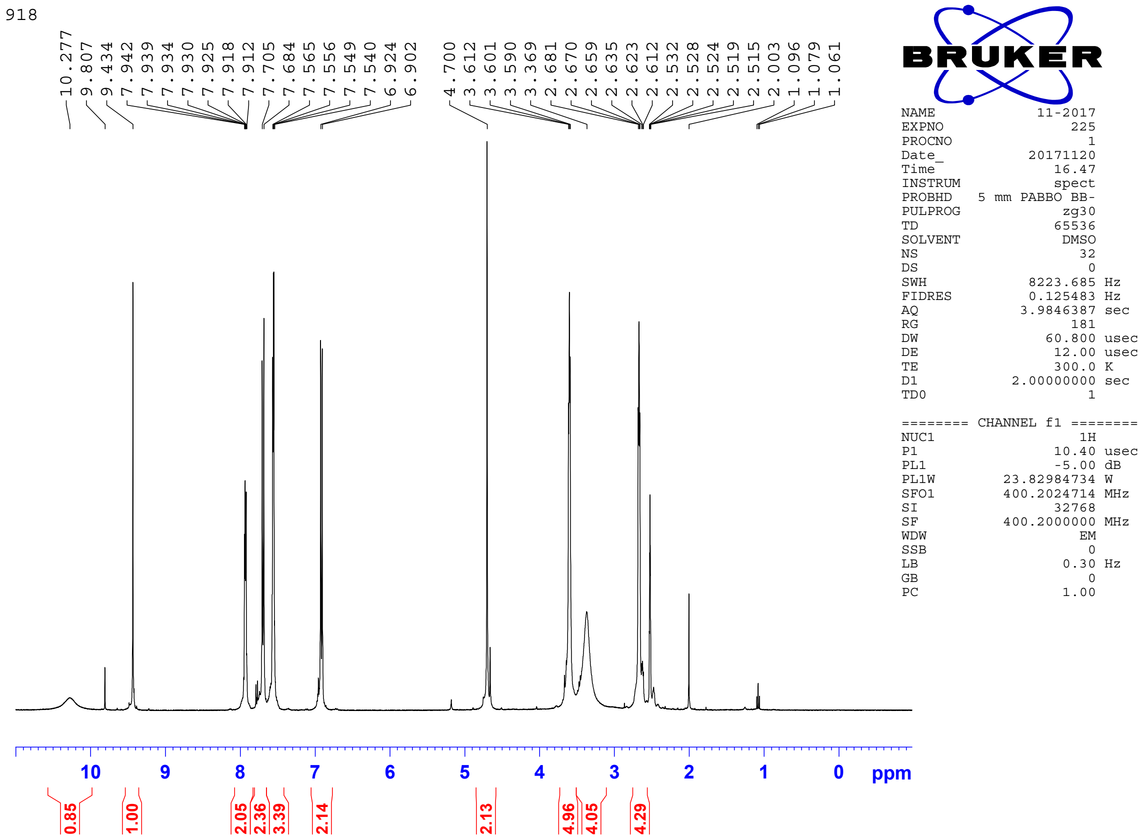

Figure S5. ^{1}H-NMR (DMS⊚ -d$_6$) Spectrum of Compound 3b

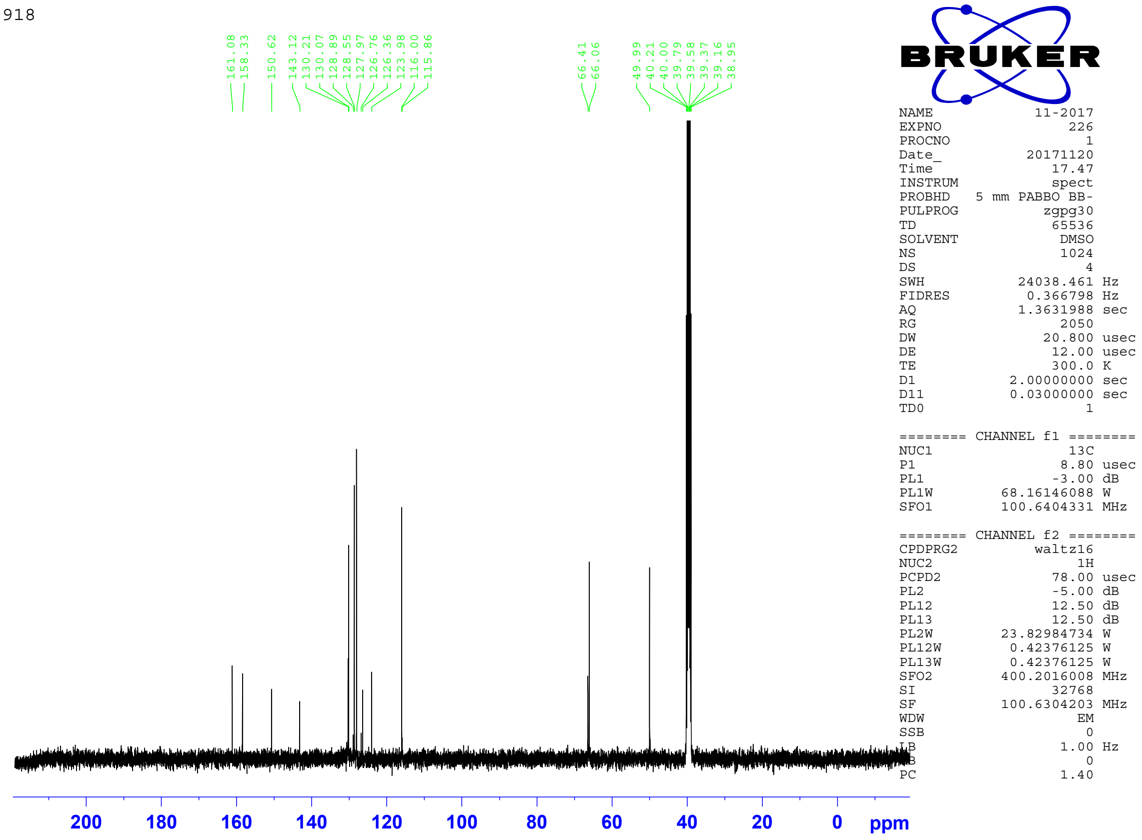

Figure S6. ^{13}C-NMR (DMS⊚ -d$_6$) Spectrum of Compound **3b**

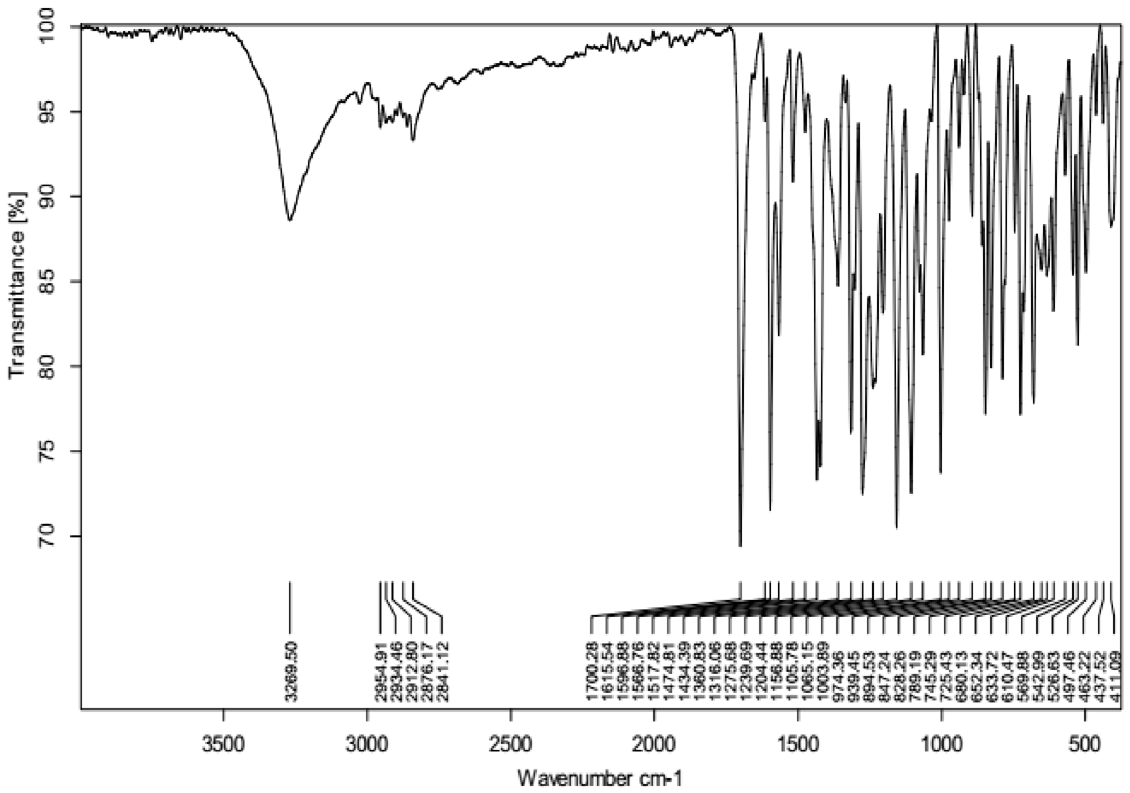

Figure S7. IR Spectrum of Compound **3c**

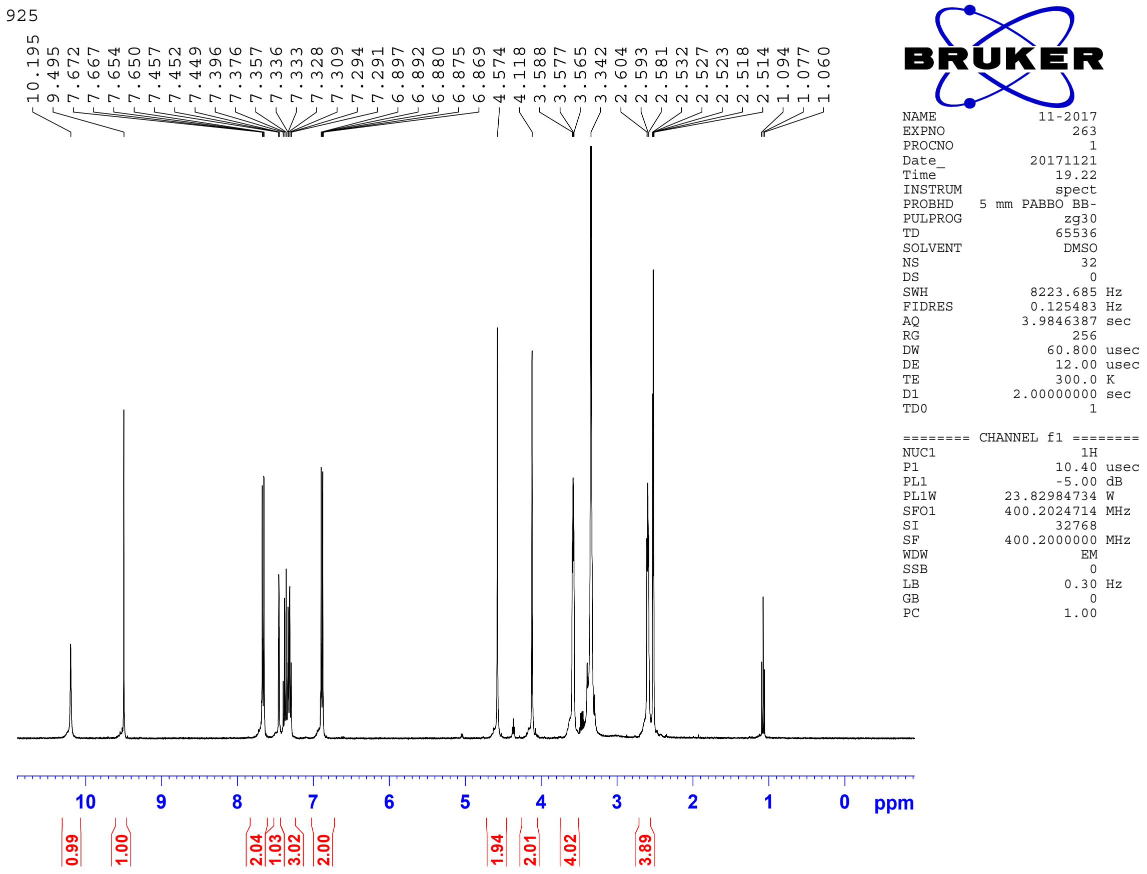

Figure S8. ¹H-NMR (DMSO -d₆) Spectrum of Compound **3c**

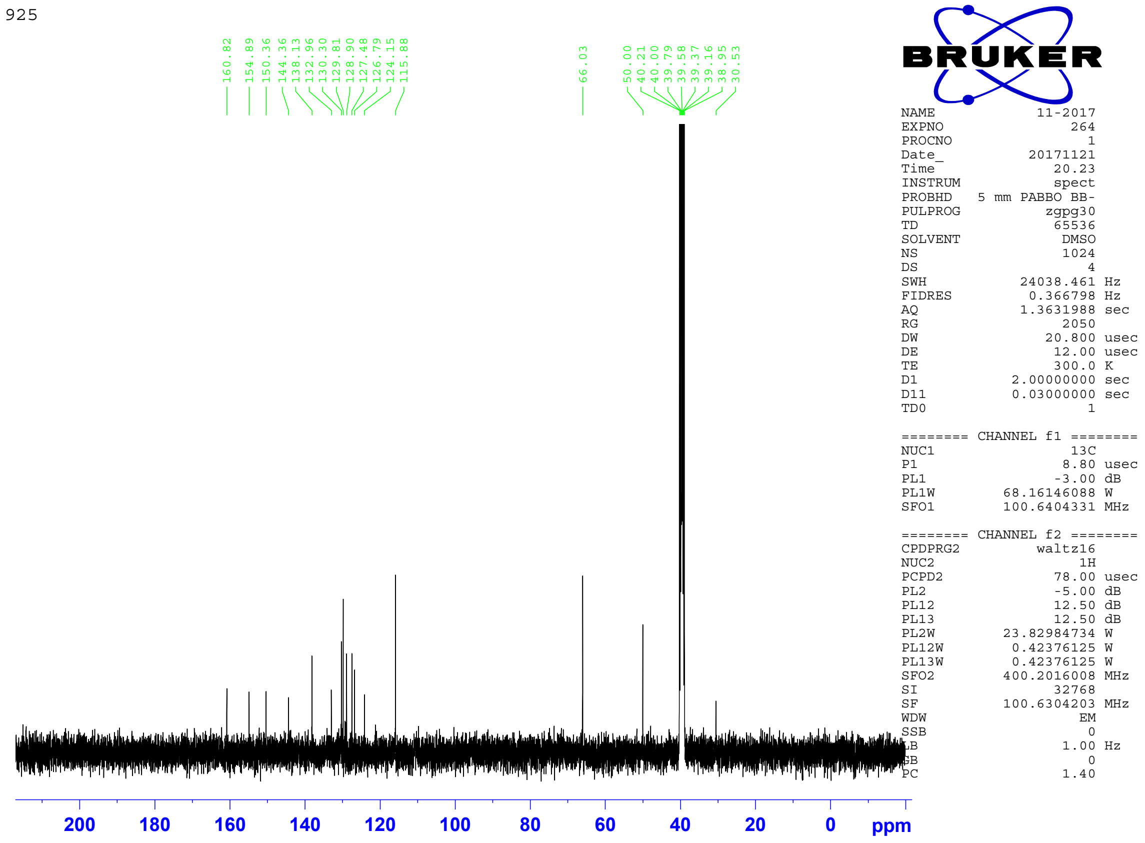

Figure S9. ^{13}C-NMR (DMS◎ -d_6) Spectrum of Compound **3c**

Table S4. Key Predicted Ligand–Protein Interactions and Bond Distances of Compound **3a** with Carbonic Anhydrase II (1BN1) and Catalase (1DGF)

Target enzyme	Interaction type	Interacting residue	Distance (Å)
**Catalase (1DGF)**	Unfavorable bump*	ARG A354	1.56
	Unfavorable bump*	ARG A354	2.00
	Unfavorable bump*	ARG A354	2.10
	π–π stacked	PHE A153	4.23
	π–π stacked	PHE A161	4.01
	Alkyl	PR◎ A158	5.02
	π–Alkyl	ARG A354	5.16
	π–Alkyl	ALA A357	3.94
	π–Alkyl	PHE A161	4.01
**Carbonic anhydrase II (1BN1)**	π–Sigma	PR◎ A202	3.47
	Amide π-stacked	PR◎ A201	4.31
	π–Alkyl	TRP A5	5.03
	π–Alkyl	HIS A64	4.83

* It represents a possible steric clash rather than a favorable interaction.

Compound **3a** interacted with catalase mainly through π–π stacked, alkyl, and π–alkyl interactions, while unfavorable contacts with ARG A354 were also observed. In carbonic anhydrase II, compound **3a** showed π–sigma, amide π-stacked, and π–alkyl interactions, without conventional hydrogen bonding.

Table S5. Key Predicted Ligand–Protein Interactions and Bond Distances of Compound **3b** with Carbonic Anhydrase II (1BN1) and Catalase (1DGF)

Target enzyme	Interaction type	Interacting residue	Distance (Å)
**Catalase (1DGF)**	Conventional hydrogen bond	ASN A148	2.67
	π–π stacked	PHE A153	4.37
	π–π stacked	PHE A161	4.08
	π–π stacked	PHE A161	5.17
	π–π stacked	TYR A358	3.81
	π–Alkyl	ARG A354	5.12
	π–Alkyl	ARG A354	5.37
	π–Alkyl	ALA A357	3.55
	π–Alkyl	ALA A357	4.81
	π–Alkyl	VAL A161*	5.30
**Carbonic anhydrase II (1BN1)**	Conventional hydrogen bond	ASN A62	2.36
	Conventional hydrogen bond	TYR A7	2.47
	Conventional hydrogen bond	GLN A92	2.18
	Carbon hydrogen bond	HIS A62*	3.34
	Carbon hydrogen bond	GLU A69	3.38
	π-Donor hydrogen bond	ASN A62	3.15
	π–Sigma	THR A200	3.95
	π–π T-shaped	HIS A94	5.05
	π–Alkyl	ALA A65	4.57

* It represents a possible steric clash rather than a favorable interaction.

Compound **3b** formed a conventional hydrogen bond with ASN A148 in catalase and showed multiple π–π stacked and π–alkyl interactions involving PHE A153, PHE A161, TYR A358, ARG A354, ALA A357, and VAL A161. In carbonic anhydrase II, compound 3b exhibited conventional hydrogen bonds with ASN A62, TYR A7, and GLN A92, together with carbon hydrogen bond, π-donor hydrogen bond, π–sigma, π–π T-shaped, and π–alkyl interactions.

Table S6. Key predicted ligand–protein interactions and bond distances of the reference inhibitors acetazolamide and 3-amino-1,2,4-triazole with carbonic anhydrase II and catalase, respectively.

Reference inhibitor	Target enzyme	Interaction type	Interacting residue	Distance (Å)
Acetazolamide	Carbonic anhydrase II (1BN1)	Conventional hydrogen bond	GLN A92	2.16
		Conventional hydrogen bond	ASN A67	2.61
		π–Sulfur	HIS A94	3.96
		π–Sulfur	TRP A5	5.01
		π–Alkyl	TRP A5	4.55
		π–Alkyl	TRP A5	5.20
3-Amino-1,2,4-triazole	Catalase (1DGF)	Conventional hydrogen bond	GLU A330	1.99
		Conventional hydrogen bond	GLY A78	2.40
		π-Donor hydrogen bond	TYR A325	2.63
		π–π T-shaped	TYR A325	5.21
		π–Alkyl	LYS A77	4.55

* Acetazolamide and 3-amino-1,2,4-triazole were used as reference inhibitors for carbonic anhydrase II and catalase, respectively. The interactions shown were obtained from the best-ranked docking poses generated using the same computational protocol applied to compounds 3a–3c.

In Tablo S6, acetazolamide exhibited two conventional hydrogen bonds with GLN A92 and ASN A67 in the carbonic anhydrase II active site, together with π–sulfur interactions involving HIS A94 and TRP A5 and π–alkyl interactions with TRP A5. In the catalase complex, 3-amino-1,2,4-triazole formed conventional hydrogen bonds with GLU A330 and GLY A78, a π-donor hydrogen bond and a π–π T-shaped interaction with TYR A325, and a π–alkyl interaction with LYS A77. These interaction profiles were derived from the best-ranked docking poses generated using the same computational protocol applied to compounds 3a–3c.

Compound **3a** was further examined in terms of its predicted binding modes and ligand–protein interaction profiles with catalase and carbonic anhydrase II (Figure S10). In catalase (1DGF), compound **3a** was positioned within the binding region mainly through π–π stacked interactions with PHE A153 and PHE A161, together with alkyl and π–alkyl interactions involving PR◎ A158, ARG A354, ALA A357, and PHE A161. Unfavorable contacts with ARG A354 were also observed, suggesting possible local steric constraints in the binding pose. In carbonic anhydrase II (1BN1), compound **3a** showed π–sigma, amide π-stacked, and π–alkyl interactions involving PR◎ A202, PR◎ A201, TRP A5, and HIS A64. No conventional hydrogen bond was observed for compound 3a in either target, which may partly explain its lower predicted binding affinity compared with compounds **3b** and **3c.**

Compound **3b** was further analyzed in terms of its predicted binding modes and ligand–protein interaction profiles with catalase and carbonic anhydrase II (Figure S11). In catalase (1DGF), compound **3b** exhibited a conventional hydrogen bond with ASN A148 and formed multiple π–π stacked interactions with PHE A153, PHE A161, and TYR A358, together with π–alkyl interactions involving ARG A354, ALA A357, and VAL A161. In carbonic anhydrase II (1BN1), compound **3b** showed several conventional hydrogen bonds with ASN A62, TYR A7, and GLN A92, as well as carbon hydrogen bond, π-donor hydrogen bond, π–sigma, π–π T-shaped, and π–alkyl interactions involving HIS A62, GLU A69, THR A200, HIS A94, and ALA A65. Compared with compound **3a**, the presence of multiple hydrogen-bonding interactions in both targets suggests that compound **3b** may adopt a more favorable binding pattern and display stronger predicted binding affinity toward these enzyme systems.

Figure S12 presents the predicted binding modes and interaction profiles of the reference inhibitors 3-amino-1,2,4-triazole and acetazolamide with catalase and carbonic anhydrase II, respectively. In the catalase complex (1DGF), 3-amino-1,2,4-triazole formed two conventional hydrogen bonds with GLU A330 and GLY A78, together with a π-donor hydrogen bond and a π–π T-shaped interaction with TYR A325, as well as a π–alkyl interaction with LYS A77. In the carbonic anhydrase II complex (1BN1), acetazolamide established conventional hydrogen bonds with GLN A92 and ASN A67, accompanied by π–sulfur interactions involving HIS A94 and TRP A5, and π–alkyl interactions with TRP A5. These interaction profiles are consistent with the predicted binding poses obtained using the same docking protocol applied to compounds 3a–3c.

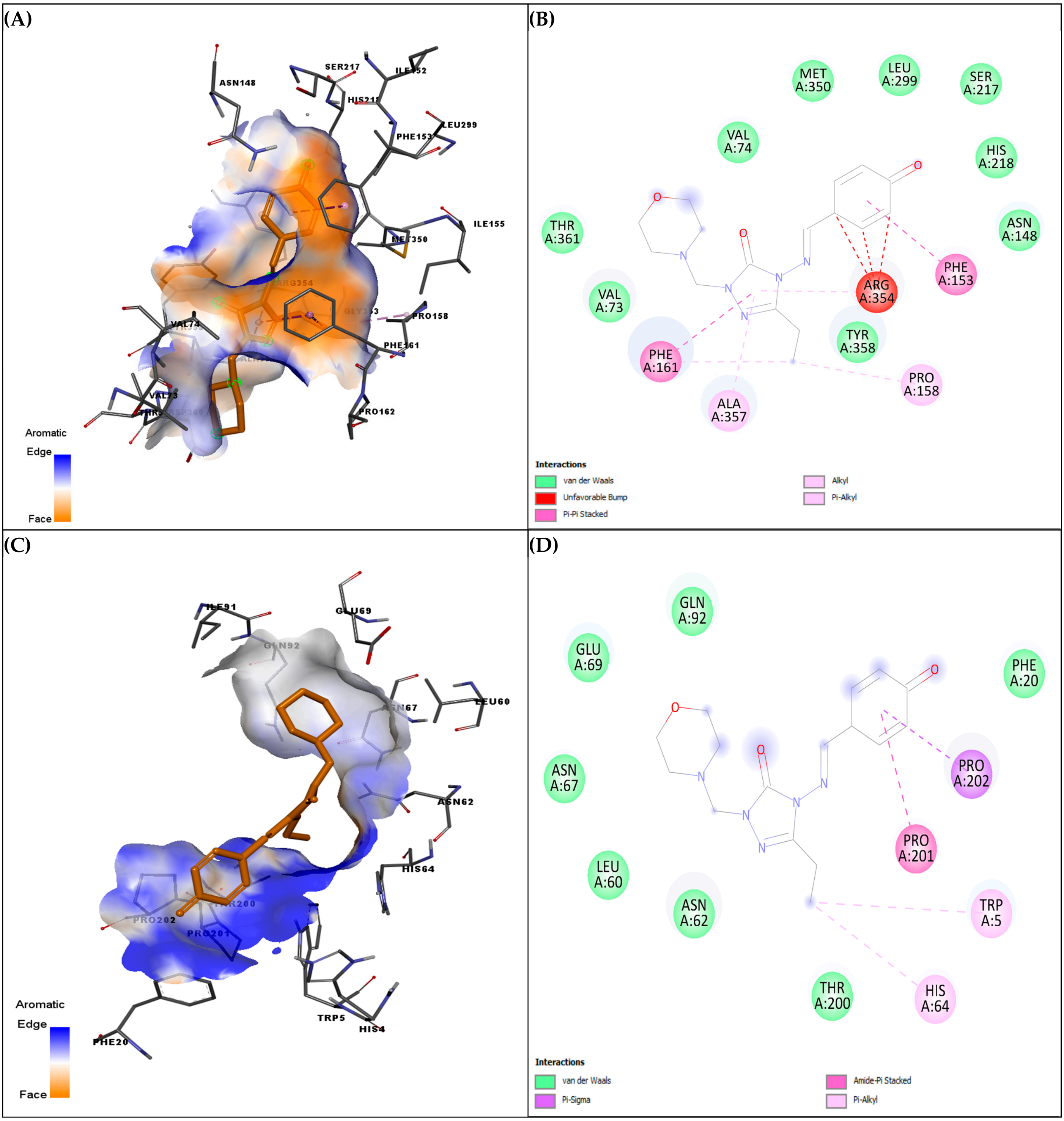

Figure S10. Predicted binding modes and interaction profiles of compound **3a** with catalase and carbonic anhydrase II. **(A)** Three-dimensional binding mode of compound **3a** in catalase (1DGF). **(B)** Two-dimensional interaction diagram of compound **3a** with catalase. **(C)** Three-dimensional binding mode of compound **3a** in carbonic anhydrase II (1BN1). **(D)** Two-dimensional interaction diagram of compound **3a** with carbonic anhydrase II.

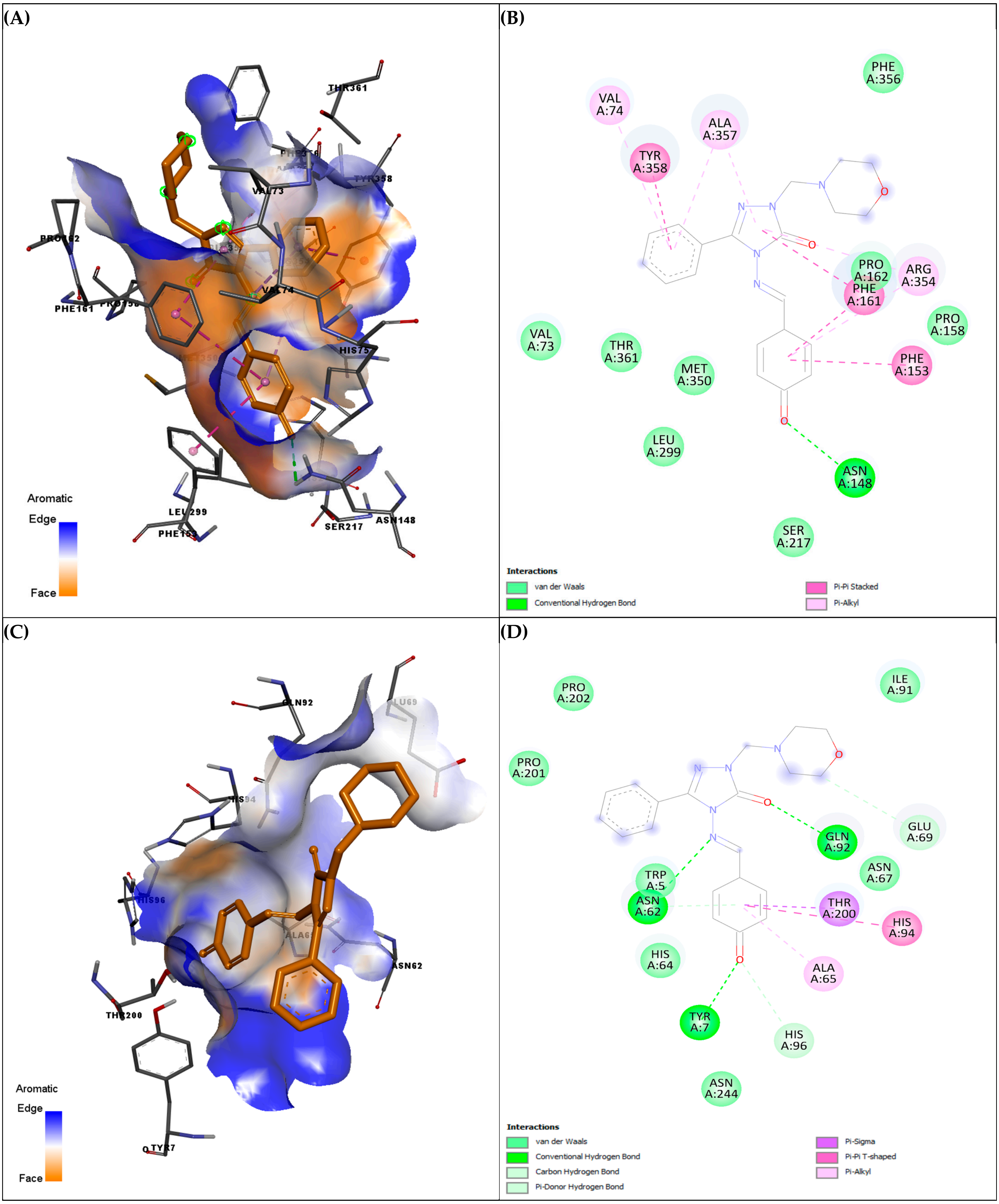

Figure S11. Predicted binding modes and interaction profiles of compound **3b** with catalase and carbonic anhydrase II. **(A)** Three-dimensional binding mode of compound **3b** in catalase (1DGF). **(B)** Two-dimensional interaction diagram of compound **3b** with catalase. **(C)** Three-dimensional binding mode of compound **3b** in carbonic anhydrase II (1BN1). **(D)** Two-dimensional interaction diagram of compound **3b** with carbonic anhydrase II.

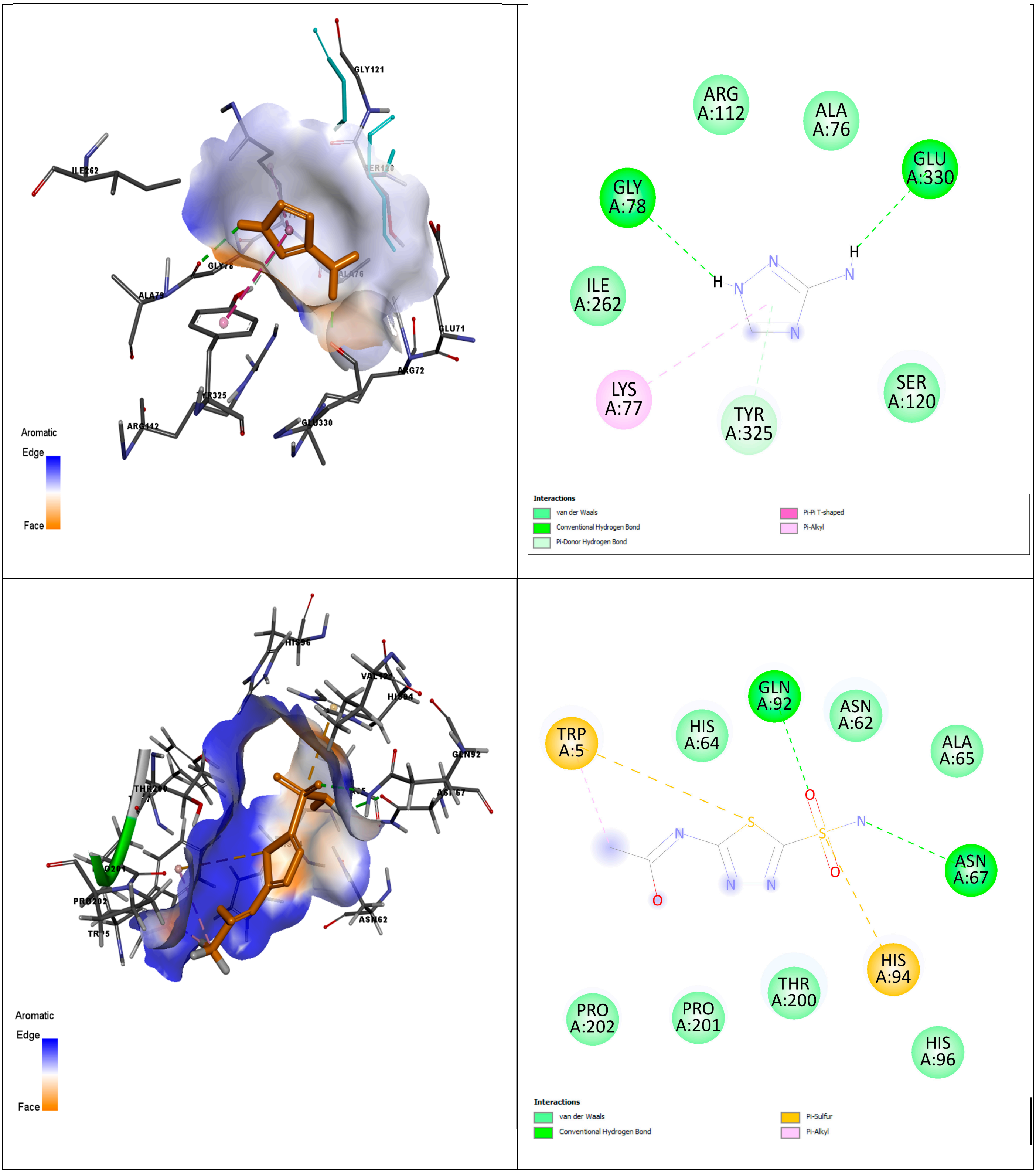

Figure S12. Predicted binding modes and interaction profiles of the reference inhibitors 3-amino-1,2,4-triazole and acetazolamide with catalase and carbonic anhydrase II, respectively. **(A)** Three-dimensional binding mode of 3-amino-1,2,4-triazole in catalase (1DGF). **(B)** Two-dimensional interaction diagram of 3-amino-1,2,4-triazole with catalase. **(C)** Three-dimensional binding mode of acetazolamide in carbonic anhydrase II (1BN1). **(D)** Two-dimensional interaction diagram of acetazolamide with carbonic anhydrase II.
